# Genetic Inhibition of Solute-Linked Carrier 39 Family Transporter 1 Ameliorates Aβ Pathology in a *Drosophila* Model of Alzheimer's Disease

**DOI:** 10.1371/journal.pgen.1002683

**Published:** 2012-04-26

**Authors:** Minglin Lang, Lei Wang, Qiangwang Fan, Guiran Xiao, Xiaoxi Wang, Yi Zhong, Bing Zhou

**Affiliations:** 1 State Key Laboratory of Biomembrane and Membrane Biotechnology, School of Life Sciences, Tsinghua University, Beijing, China; 2 College of Life Science, Agricultural University of Hebei, Baoding, China; 3 Department of Biochemistry, Kansas State University, Manhattan, Kansas, United States of America; 4 Cold Spring Harbor Laboratory, Cold Spring Harbor, New York, United States of America; Stanford University School of Medicine, United States of America

## Abstract

The aggregation or oligomerization of amyloid-β (Aβ) peptide is thought to be the primary causative event in the pathogenesis of Alzheimer's disease (AD). Considerable *in vitro* evidence indicates that the aggregation/oligomerization of Aβ is promoted in the presence of Zn; however, the functional role of Zn in AD pathogenesis is still not well clarified *in vivo*. Zn is imported into the brain mainly through the solute-linked carrier (*Slc*) 39 family transporters. Using a genetically tractable *Drosophila* model, we found that the expression of *dZip1*, the orthologue of human *Slc39* family transporter *hZip1* in *Drosophila*, was altered in the brains of Aβ42-expressing flies, and Zn homeostasis could be modulated by forcible *dZip1* expression changes. An array of phenotypes associated with Aβ expression could be modified by altering *dZip1* expression. Importantly, Aβ42 fibril deposits as well as its SDS-soluble form were dramatically reduced upon *dZip1* inhibition, resulting in less neurodegeneration, significantly improved cognitive performance, and prolonged lifespan of the Aβ42-transgenic flies. These findings suggest that zinc contributes significantly to the Aβ pathology, and manipulation of zinc transporters in AD brains may provide a novel therapeutic strategy.

## Author Summary

Alzheimer's disease (AD) is characterized by extracellular amyloid plaques and altered metal ion (including Zn, Cu, Fe) concentrations in the brain. Amyloid plaques are the result of increased aggregation of Aβ, while the *in vivo* role of metal ions such as Zn remains poorly understood. We found that the expression of a zinc transporter (*dZip1*) is altered in the brains of AD flies. Genetic manipulation of *dZip1* to modulate its expression was accompanied by altered Aβ accumulation, resulting in changes in the neurodegeneration development, cognitive performance, and lifespan of the AD flies. These genetic findings support the zinc role in AD pathology and implicate a new therapeutic target for treating AD.

## Introduction

Alzheimer's disease (AD) is a major neurodegenerative disease affecting the elderly. The accumulation of amyloid-β (Aβ) peptides, which either form the major component of senile plaques (SP) or the oligomer state in patient brains, is hypothesized to be the primary causative event in AD pathogenesis [1]–[3]. However, what drives the Aβ accumulation and how this accumulation links to progression of the disease is not well understood.

Increasing evidence indicates that the disruption of metal homeostasis, particularly in Zn and Cu concentrations, is strongly correlated with the pathophysiological process of AD [4]–[6]. Although copper and, to a lesser extent, iron can induce partial Aβ aggregation, they need a mildly acidic condition (pH 6.6) [7]. Zn^2+^ is the only metal ion available to aggregate Aβ at pH7.4-the normal physiological pH [8]–[10]. Elevated Zn was found and co-purified with Aβ from AD brain tissues, associated with markedly high Zn level in cerebral spinal fluid (SP) [11]. Measurements from well characterized late stage AD (LAD) also showed a significant increase of Zn in brain sections of hippocampus, multiple neocortical areas and amygdala compared to age-matched normal control subjects [4], [5], [12]–[14]. Although several reports indicate that Zn induces Aβ aggregation at low physiological concentrations [8], [15], [16], later studies showed that higher Zn concentrations are required for significant fibril formation [17], [18]. These pieces of evidence were obtained mostly from *in vitro* experiments. Therefore how Zn status influences Aβ pathology *in vivo* throughout life course remains unclear. Dietary intervention of zinc intake with zinc chelators in animals show some encouraging results [19], [20], [21]. However, genetic evidence is still lacking. More importantly, zinc chelators are usually not zinc specific, and may associate with other nonspecific phenotypes [22], precluding accurate mechanism analysis.

Transport of Zn into cells is mediated by a set of zinc transporters called Zrt-Irt like proteins (Zips). Zips are characterized as influx transporters that mediate Zn^2+^ uptake into cytoplasm from extracellular or vesicular sources [23], [24], and are encoded by the solute-linked carrier (*Slc*) gene family, *Slc39* [23]. At least 14 Zips have been identified in the human genome [23] and 8 in *Drosophila* [25]. Most Zips are predicted to have eight transmembrane domains (TM) with a histidine-rich loop between TM3 and TM4, and to be located at the plasma membrane [26]. Although the uptake of Zn from the brain's extracellular environment to intracellular compartments in neurons and glia cells is not completely understood, the Zips are thought to be involved in this process [23], [24]. To our knowledge, the relationship between Zips and AD has not been explored to date.

Previously we and others have shown that expression of human Aβ42 in *Drosophila* brains recapitulates the main symptoms of AD including age-dependent memory loss, formation of amyloid deposits and neurodegeneration [27], [28]. In the current study, we found that the time course expression change of *dZip1*, an orthologue of human *Slc39* family transporter *hZIP1* in *Drosophila*, was reversed in brains of Aβ42 flies as compared with normal control flies. We hypothesize that modulating dZip1 expression level might affect Zn accumulation in the brain and modify the AD pathological process. By creating *dZIP* overexpression and RNAi transgenic flies, we demonstrated that dZip1 is critically involved in Aβ-induced AD pathological process, and by lowering dZip1 expression Aβ toxicity can be markedly ameliorated.

## Results

### dZip1 is a putative Zn importer involved in zinc uptake

**Figure 1:**
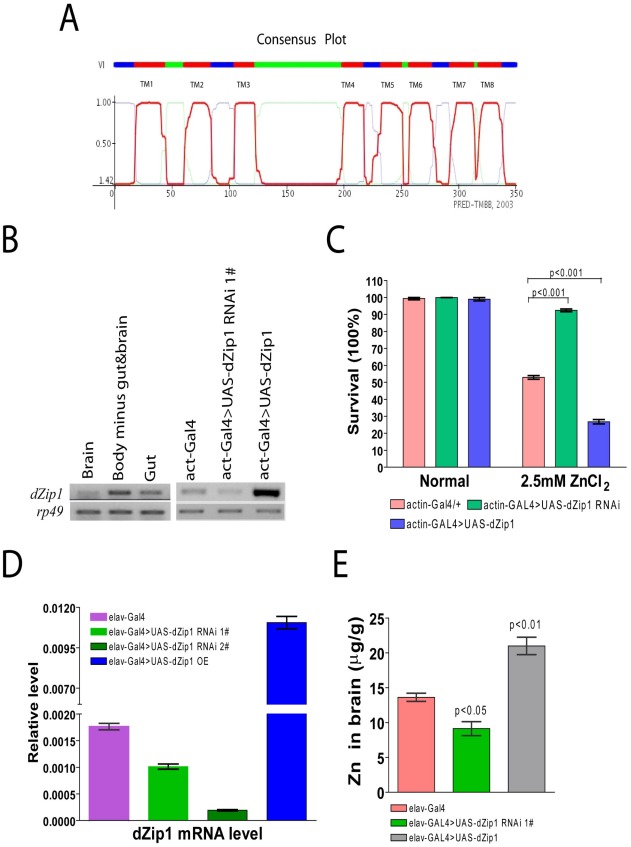
*dZip1* expression modulation result alteration in Zn sensitivity and Zn accumulation in *Drosophila*. (A) dZip1 protein belongs to the *Drosophila Slc39* family with eight typical conserved transmembrane domains. The plot shows HMM-TM software predicted dZip1 transmembrane alpha-helicals, which includes eight transmembrane domains (TM 1 to 8) and a long His-rich loop (between TM 3 and 4) inside the cytosol. The N- and C-terminals were predicted to be located outside of the cytosol. (B) *dZip1* expression analysis. On the left shows *dZip1*expression in the brain, gut and whole body minus gut & brain of *w^1118^* adult flies, and on the right *dZip1* expression in the whole body of *actin-Gal4*, *actin-Gal4>UAS-dZip1-RNAi 1#* and *actin-Gal4>UAS-dZip1* adult flies (right). Expression analysis was performed with sqRT-PCR, with *rp49* as the loading control. (C) *dZip1* expression change leads to Zn sensitivity change. Three-day-old flies were exposed to 2.5 mM ZnCl_2_ or no drug, *dZip1* ubiquitous expression flies (*actin-Gal4>UAS-dZip1*) were significantly more sensitive to 2.5 mM ZnCl_2_ treatment than the control flies (*actin-Gal4*) (*t*-test, p<0.001). Inhibiting *dZip1* expression (*actin-Gal4>UAS-dZip1-RNAi 1#*) significantly enhanced flies' tolerance against 2.5 mM ZnCl_2_ treatment (*t* test, p<0.001). No significant differences were found among all the genotypes when raised on normal food. (D) *dZip1* mRNA level can be changed in fly brains by *dZip1* RNAi and over-expression when using a pan-neuronal elav-Gal4 driver. Compared with control *elav-Gal4* flies, brain *dZip1* transcript level was repressed nearly twofold in *elav-Gal4>UAS-dZip1-RNAi 1#* transgenic flies, and was repressed approximately ten fold in *elav-Gal4>UAS-dZip1-RNAi 2#* transgenic flies, while it was approximately sevenfold over-expressed in *elav-Gal4>UAS-dZip1* transgenic flies. The relative *dZip1* expression levels against *rp49* were from three independent biological replicates of each genotype and plotted with SEM (error bars). (E) Inductively-coupled-plasma-optical-emission-spectrometry (ICP-OES) was used to detect the Zn content in 15 day old fly brains. Pan-neuronal expression of *dZip1* (*elav-Gal4>UAS-dZip1*) increased the Zn level (*t*-test, P<0.01) while *dZip1* RNAi (*elav-Gal4>UAS-dZip1-RNAi 1#*) reduced Zn accumulation (*t*-test, P<0.05).

Using the amino acid sequence of hZip1, BLAST searches revealed 8 putative Zips in *Drosophila*, among which *CG9428*-encoded putative protein shared the highest similarity with human Zip1 (29% identity). We designate it as dZip1. Topology analysis of dZip1 revealed the presence of eight putative transmembrane domains, a histidine–rich loop between domains 3 and 4 which was predicted to occur within the cytosol, and extracellular N- and C-terminals (Figure 1A). All these features are typical of *Slc39* family members [23], [24].

We measured *dZip1* transcript levels in organs of *w^1118^* flies by semi-quantitative RT-PCR (sqRT-PCR). *dZip1* is expressed in the gut and other organs including the brain (Figure 1B left). To further explore the physiological role of dZip1 in flies, we created *dZip1* over-expression (OE) and RNAi transgenic flies. We confirmed elevated and decreased *dZip1* transcript levels accordingly by *dZip1* over-expression or RNAi in whole body of transgenic flies driven by actin-Gal4 (Figure 1B Right). We then tested the zinc sensitivity of these flies. Figure 1C shows that *dZip1* OE flies were more sensitive to zinc overdose (*t*-test, p<0.001), while *dZip1*-*RNAi* flies were more tolerant to zinc overdose (*t*-test, p<0.001) in comparison with controls. These results suggest that *dZip1* is indeed involved in zinc uptake.

To test whether brain Zn levels could be changed accordingly by specifically modulating brain dZip1 level, the pan-neuronal elav-Gal4 driver was used to drive *dZip1* OE and RNAi in fly brains. Figure 1D showed qRT-PCR results of *dZip1* transcript level in different transgenic fly brains. Two *dZip1*-*RNAi* transgenic lines were used, in which the knockdown effect of *dZip1*-*RNAi* #2 transgenic line (∼1/10 of the *dZip1* transcript level in control *elav-Gal4* flies) was much stronger than *dZip1*-*RNAi* #1 transgenic line (∼3/5 of the *dZip1* transcript level in control *elav-Gal4* flies) (Figure 1D). *dZip1* OE transgenic line showed ∼6–7 fold increase of *dZip1* transcript level compared with control brains (Figure 1D). Inductively coupled plasma optical emission spectrometry (ICP-OES) result showed that over-expression of *dZip1* in the fly brain markedly increased brain Zn accumulation (*t*-test, P<0.01), while knocking down *dZip1* via RNAi decreased brain Zn level compared with the control *elav-Gal4* flies (*t*-test, p<0.05) (Figure 1E). Therefore, specific manipulation of *dZip1* expression level in fly brains could affect the brain Zn status.

### Aβ42 affects *dZip1* expression pattern in the course of aging

**Figure 2:**
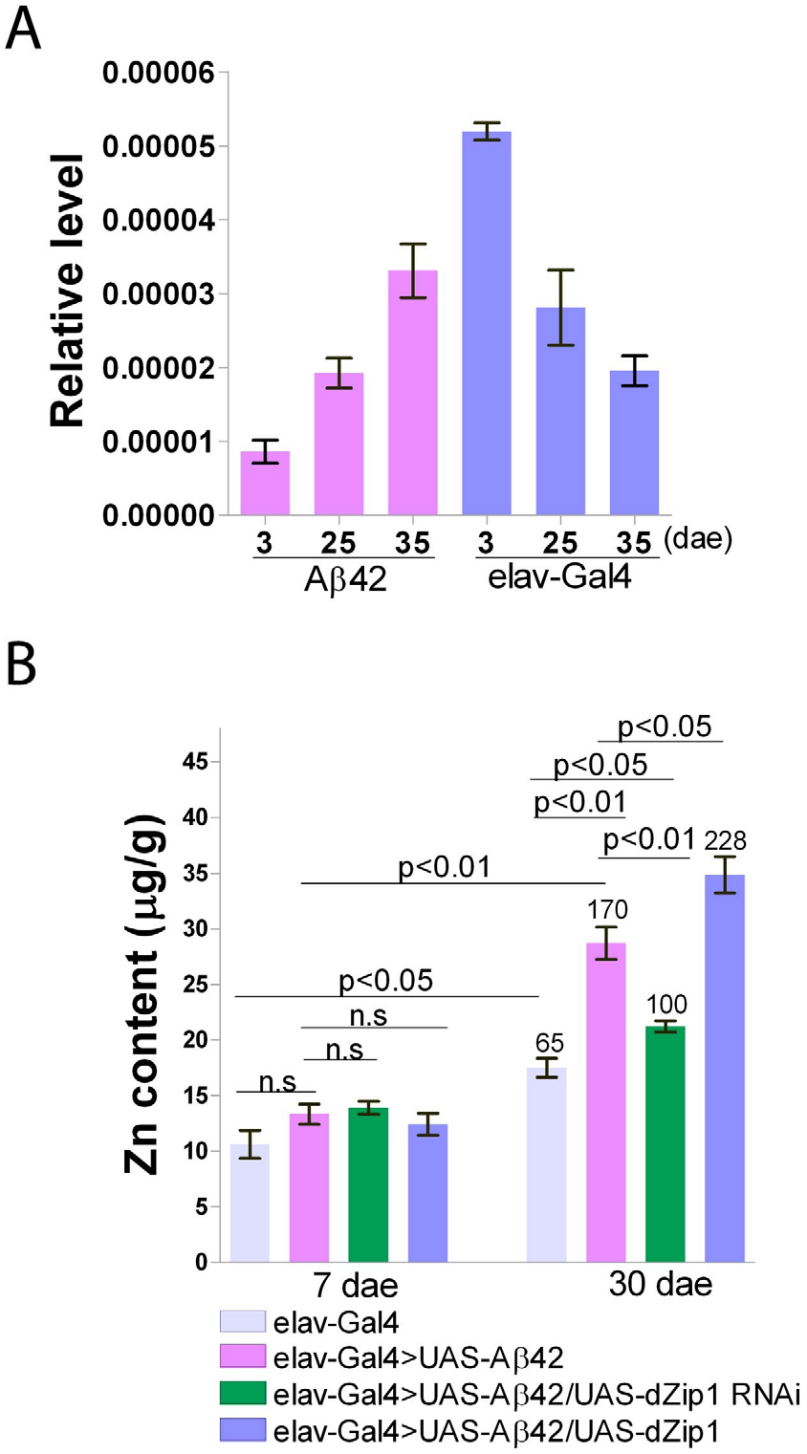
Brain *dZip1* expression and zinc levels are affected by Aβ42 expression and aging. (A) *dZip1* expression *vs*. Aβ42 expression and aging. qRT-PCR was used to test *dZip1* mRNA levels in brains of different ages of *elav-Gal4* and *elav-Gal4>UAS-Aβ42* (Aβ42) flies. *dZip1* transcript level increased in brains of Aβ42 flies, but decreased in brains of *elav-Gal4* flies with ageing. In 2-3-day old early enclosed fly brains, *dZip1* transcript level of *elav-Gal4* flies was approximately six fold greater than that of the age-matched Aβ42 flies. The relative *dZip1* expression levels against *rp49* are from three independent biological replicates of each genotype and plotted with SEM (error bars). (B) Brain zinc levels *vs*. Aβ42, *dZip1* expression and aging. Zn content in brains of 7- and 30-day old flies was measured by ICP-OES. *dZip1* OE (*elav-Gal4>UAS-Aβ42/UAS-dZip1*) facilitated Zn accumulation in fly brains compared with control *elav-Gal4* and Aβ42 flies (*elav-Gal4>UAS-Aβ42*). *dZip1* RNAi (*elav-Gal4>UAS-Aβ42/UAS-dZip1-RNAi*) slowed down the brain Zn accumulation process. Phenotypes are more obvious in 30-day old flies. Data on top of the “30 dae” bars represent the relative increased percentage of Zn level to 7-day old control elav-Gal4 flies. Data are expressed as means ± SEM and analyzed by Student's *t*-test. n = 3 for each genotype.

Aβ42 expression in fly brains could induce an age-dependent formation of amyloid deposits and neurodegeneration which may correlate with disturbed metal homeostasis, especially for zinc and copper. We therefore checked native *dZip1* mRNA levels in brains of *elav-Gal4*>*UAS-Aβ42* (Aβ42) flies and normal *elav-Gal4* flies at different ages. We found that the *dZip1* mRNA level was developmentally altered in Aβ42 fly brains compared to *elav-Gal4* fly brains (Figure 2A). The *dZip1* mRNA level in brains of *w^1118^* flies showed similar results as *elav-Gal4* flies (data not shown), indicating that this is not due to the effect of the introduced *Gal4* gene. These results imply that Aβ expression may indeed lead to a zinc dyshomeostasis in the fly brains. Of note is that the endogenous *dZip1* expression was lower in young adult Aβ flies as compared to the control, although the brain zinc level of the Aβ flies at the young stage was not significantly different (Figure 2B), suggesting a complex regulation of zinc metabolism (involving participants such as other zinc importers and exporters besides *dZip1*) is involved in the brain zinc control.

Using ICP-OES we directly measured the Zn content in brains of 7- and 30-day old flies. By 7 days of age, differences of Zn content among different groups were still not apparent (Figure 2B). With ageing, the Zn content in all the brains significantly increased; however, in comparison with 7-day old *elav-Gal4* flies without Aβ expression, 30-day old Aβ42 flies increased ∼170% of their Zn level, significantly higher than that of 30-day old *elav-Gal4* flies (average ∼65% increase of the control Zn). Over-expression of *dZip1* further increased Zn accumulation in Aβ42 fly brains, while *dZip1* knockdown slowed brain Zn accumulation during aging and significantly reduced Zn accumulation compared to 30-day old Aβ42 flies (*t*-test, p<0.01). By testing Zn content of these flies at 20-day old (Figure S1), although it's not obvious as that of 30-day, the trend is already apparent and statistically significant. These results indicate an intimate connection among Aβ42 expression, aging and brain zinc accumulation, and the latter can be strongly affected by dZip1 expression interference.

### Modulation of *dZip1* expression correspondingly alters the course of Aβ42-induced neurodegeneration

**Figure 3:**
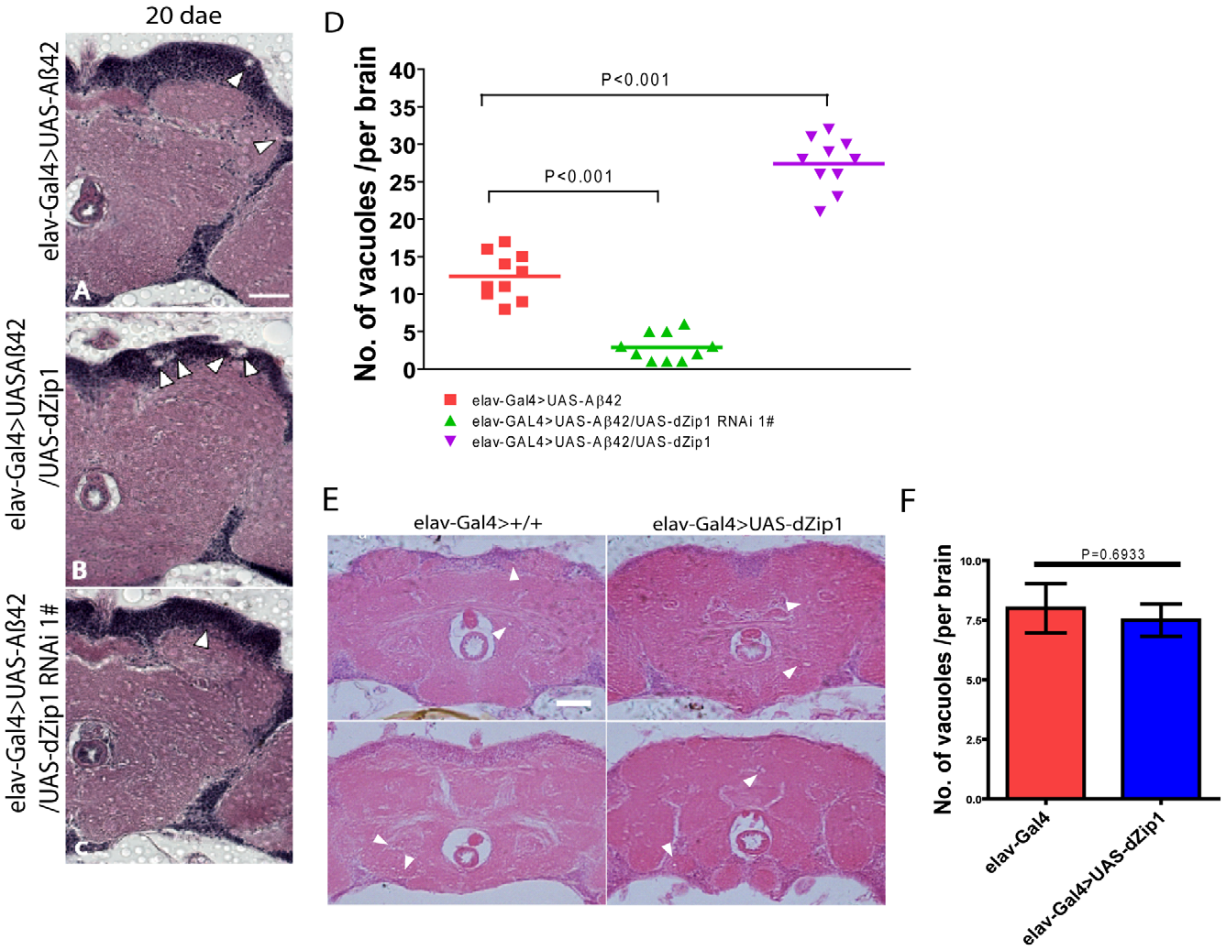
Aβ42-induced neurodegeneration is ameliorated by *dZip1* RNAi but exacerbated by *dZip1* overexpression. Paraffin sections of 20- or 30-day old fly brains were stained with H&E (A–C). Pan-neuronal expression of Aβ42 in fly brains (*elav-Gal4>UAS-Aβ42*) induced neurodegeneration (arrowheads indicate the vacuoles) in both the cortex and the neuropil region. (B) shows that *dZip1* OE produced more vacuoles than the age-matched control (A), while *dZip1* RNAi ameliorated neurodegeneration (C). (D) is a statistical analysis of Aβ42-induced neurodegeneration under *dZip1* expression modulation. Brain sections across the mushroom body somatic region were chosen for comparison. Number of vacuoles (diameter>3 µm) on each section was counted and summarized. Significant differences were seen between *dZip1* OE (*elav-Gal4>UAS-Aβ42/UAS-dZip1*) flies and controls (*elav-Gal4>UAS-Aβ42*) (p<0.001), and between *dZip1*-*RNAi* (*elav-Gal4>UAS-Aβ42/UAS-dZip1-RNAi*) flies and controls (p<0.001). (E) shows that *dZip1* overexpression alone does not lead to significant neurodegeneration. Paraffin sections of 20-day old *elav-Gal4* and *elav-Gal4>UAS-dZip1* fly brains were stained with H&E. Pan-neuronal expression of *dZip1* in fly brains did not induce significant neurodegeneration in both the cortex and the neuropil region compared to *elav-Gal4* flies. Vacuoles indicated by arrowheads. (F) is a statistical analysis of vacuoles (diameter >1 µm) in *elav-Gal4* and *elav-Gal4>UAS-dZip1* fly brain sections. Data are expressed as means ± SEM and analyzed by the Student's *t*-test. n = 6–10 for each genotype. Scale bar, 50 µm.

Next, we used Hematoxylin and Eosin (H&E) staining to examine whether the extent of brain neurodegeneration in aged Aβ42 flies (visualized as vacuolization in the brain region, arrowhead in Figure 3) could be changed by modulation of *dZip1* expression. Compared to the control, Aβ42-expressing brains with *dZip1* knockdown were to a large extent normal (Figure 3C), but when *dZip1* was overexpressed degenerative changes were significantly more apparent in both the cortex and the neuropil region, where there were more and bigger bubbles (Figure 3B). Counting the number of vacuoles in the cortex and neuropil revealed that over-expression of *dZip1* increased brain vacuolization more than 2-fold (Figure 3D, *t*-test, p<0.001), whereas *dZip1* knockdown dramatically decreased brain vacuolization in Aβ42 flies (*t*-test, p<0.001). Meanwhile, no significant different of neurodegenerative bubbles were found between *dZip1 OE* alone and age-matched control *elav-Gal4* flies in 20-day old fly brains (Figure 3E and 3F). To exclude possible off-target effect of the RNAi action, we confirmed the results with a differently constructed RNAi line, V3986, from the VDRC stock center. V3986 exhibited a similar level of *dZip1* reduction as our own *dZip1-RNAi* line #2 (Figure S2), and could roughly to the same extent rescue Aβ-associated brain vacuolization (Figure S2C and S2D). These results indicated that modulating *dZip1* expression could change the brain neurodegenerative process.

### Modulation of dZip1 expression level affects the climbing ability and lifespan of Aβ42 flies

**Figure 4:**
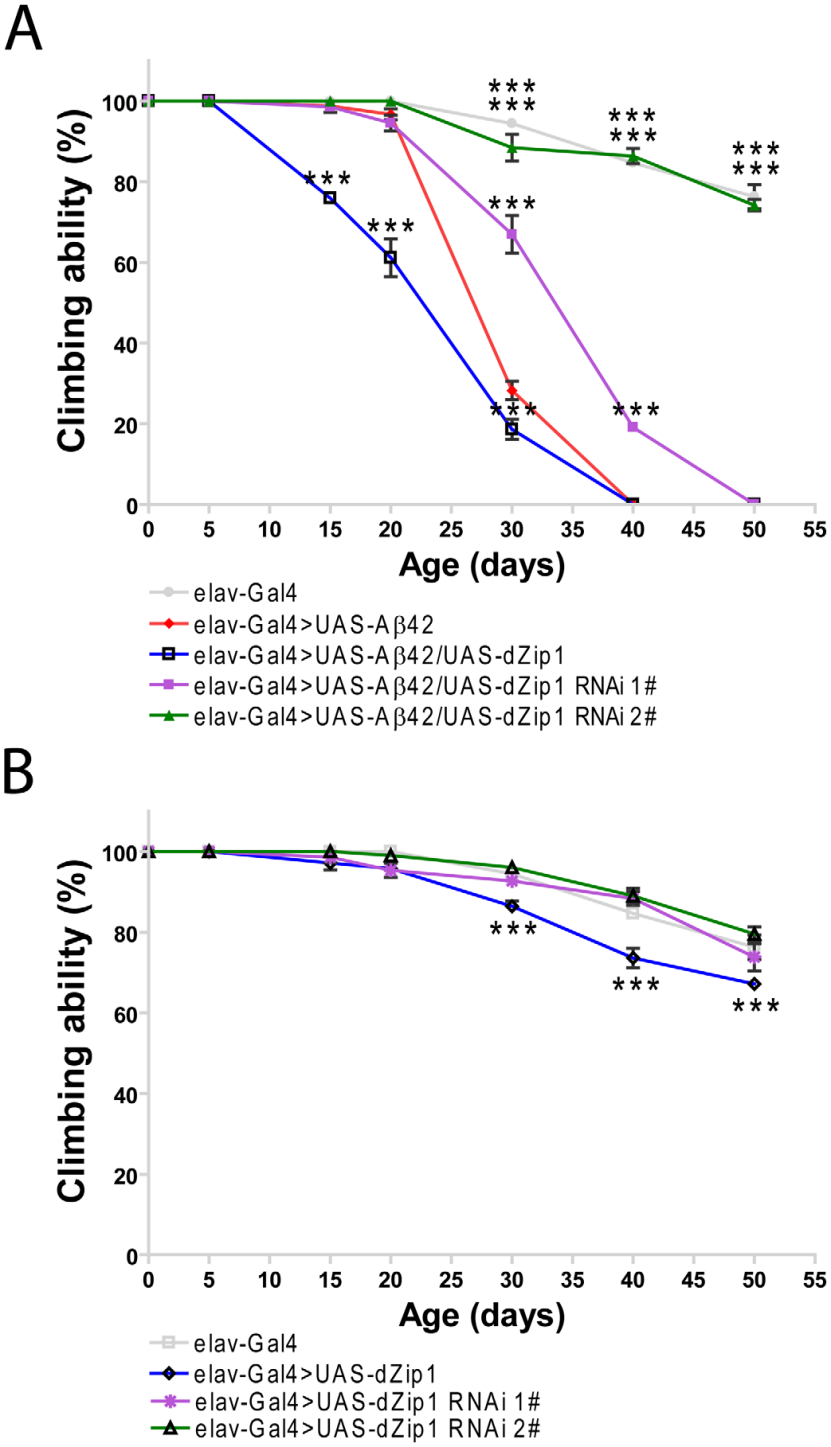
*dZip1* expression reduction rescues the climbing defect of Aβ42 flies. (A) Aβ42 expression in fly brains (*elav-Gal4>UAS-Aβ42*) induced a climbing deficit as compared with the control flies (*elav-Gal4*). *dZip1* OE (*elav-Gal4>UAS-Aβ42/UAS-dZip1*) enhanced the Aβ42-induced climbing defect. *dZip1*-*RNAi* (*elav-Gal4>UAS-Aβ42/UAS-dZip1-RNAi*) inhibited Aβ42 toxicity with a dose-dependent manner in that *dZip1-RNAi #2* exhibited a more significant rescue. *t*-test, **P<0.01, ***P<0.001 (in comparison with *elav-Gal4>UAS-Aβ42* flies). n = 4 independent experiments. (B) In the absence of Aβ42 expression, no significant locomotor deficits were found among *elav-Gal4*, *elav-Gal4>UAS-dZip1-RNAi 1#* and *elav-Gal4*>*UAS-dZip1-RNAi 2#* flies, but *elav-Gal4>UAS-dZip1* flies displayed noticeable locomotor deficits at 30 days after eclosion (dae). *t-*test, **P<0.01, ***P<0.001 (in comparison with *elav-Gal4* flies). n = 4 independent experiments.

It has been shown that Aβ42 flies start to display locomotor dysfunction after three weeks of age and their lifespan is significantly reduced [27], [28]. We therefore tried to examine whether *dZip1* expression levels could affect Aβ42 flies' locomotion and lifespan. Two *dZip1*-*RNAi* transgenic lines were used, in which the knocking down effect of the *dZip1*-*RNAi #2* transgenic line was much more obvious than the *dZip1*-*RNAi #1* transgenic line (Figure 1D). Assay of climbing ability demonstrated that Aβ42 flies with *dZip1* overexpression started to have a locomotor defect at 15-day old age comparable with that of the control *elav-Gal4>UAS-Aβ42* flies at between 20–25 days (Figure 4A). In contrast, Aβ42 flies with decreased *dZip1* levels through RNAi (#1) had a delayed climbing deficit (Figure 4A). This rescuing effect on climbing ability was even more pronounced when the stronger line *dZip1-RNAi #2* was used. As a further control, we tested the climbing ability of the transgenic flies without Aβ42 expression (Figure 4B). Only flies with over-expression of *dZip1* manifested mild locomotor defect 30 days after eclosion as compared with the age-matched control *elav-Gal4* flies. Again we confirmed the climbing rescuing effect with a different RNAi line V3986 and found it compared similarly with our *dZip1-RNAi* flies (Figure S2E). We conclude the locomotion defect as a result of Aβ42 toxicity can be modulated through the change of *dZip1* expression level.

**Figure 5:**
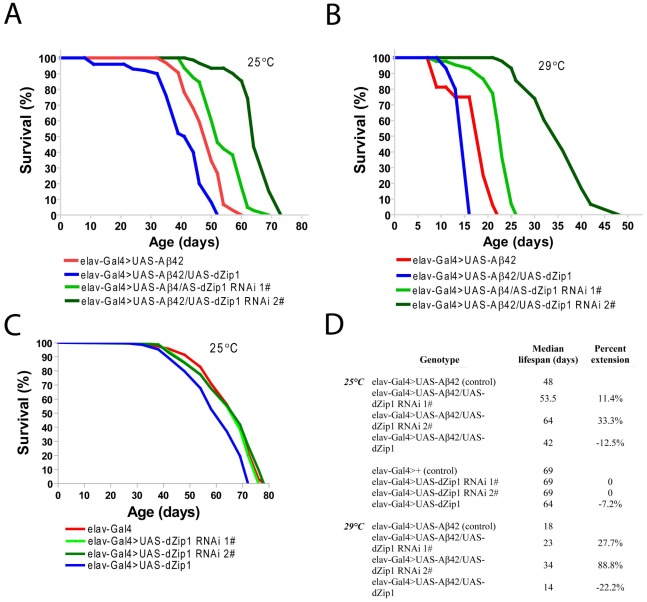
*dZip1* knockdown significantly lengthens the lifespan of Aβ42 flies. The percentage of survivorship was plotted against the age (dae). *dZip1* OE significantly shortened the life span of Aβ42 (*elav-Gal4>UAS-Aβ42*) flies with a 12.5% and a 22.2% reduction in the median lifespan reared at 25°C (p<0.001, A and D) and 29°C (p<0.001, B and D), respectively. *dZip1* RNAi significantly prolonged the life span of *elav-Gal4>UAS-Aβ42* flies, in which *dZip1-RNAi 1#* (*elav-Gal4>UAS-Aβ42/UAS-dZip1-RNAi 1#*) flies had a 11.4% and 27.7% increase in the median lifespan over that of the Aβ42 flies at 25°C (p<0.001, A and D) and 29°C (p<0.001, B and D), respectively, and *dZip1*-*RNAi* 2# (*elav-Gal4>UAS-Aβ42/UAS-dZip1-RNAi 2#*) flies had a 33.3% and a 88.8% increase in the median lifespan over that of the Aβ42 flies at 25°C (p<0.001, A and D) and 29°C (p<0.001, B and D), respectively. Without Aβ42 expression, the median lifespans of *dZip1-RNAi 1#* and *2#* flies were the same as that of the control *elav-Gal4* flies (C and D), but that of the *dZip1* OE flies showed a 7.2% reduction (p<0.001, C and D) when raised at 25°C. Differences shown are all statistically significant (p<0.001). Reported P values are from Mantel-Cox log-rank statistical analysis.

Consistent with the result obtained in the climbing assay, the lifespan of Aβ42 flies was shortened by over-expression of *dZip1* and prolonged by RNAi-based knockdown of *dZip1* expression (Figure 5A and 5B). The *dZip1-RNAi #2* transgenic line exhibited the strongest rescue, with 33.3% and 88.8% increase respectively in the median lifespan of Aβ42 flies reared at 25°C and 29°C (Figure 5D). Similar to that in the climbing assay, the lifespans of flies without Aβ42 expression were largely indistinguishable except for that of the dZip1 OE (*elav-Gal4>UAS-dZip1*) flies, which displayed a noticeable 7.2% reduction (Figure 5C and 5D). Our results indicate that a reduction of dZip1 expression in Aβ42 flies leads to improved locomotor ability and longer lifespan. Consistently, zinc chelation with clioquinol extended Aβ42 survival (Figure S3). Interestingly, clioquinol appeared rescuing male Aβ42 more effectively than females.

### Modulation of *dZip1* expression ameliorates Aβ42-induced early memory loss

**Figure 6:**
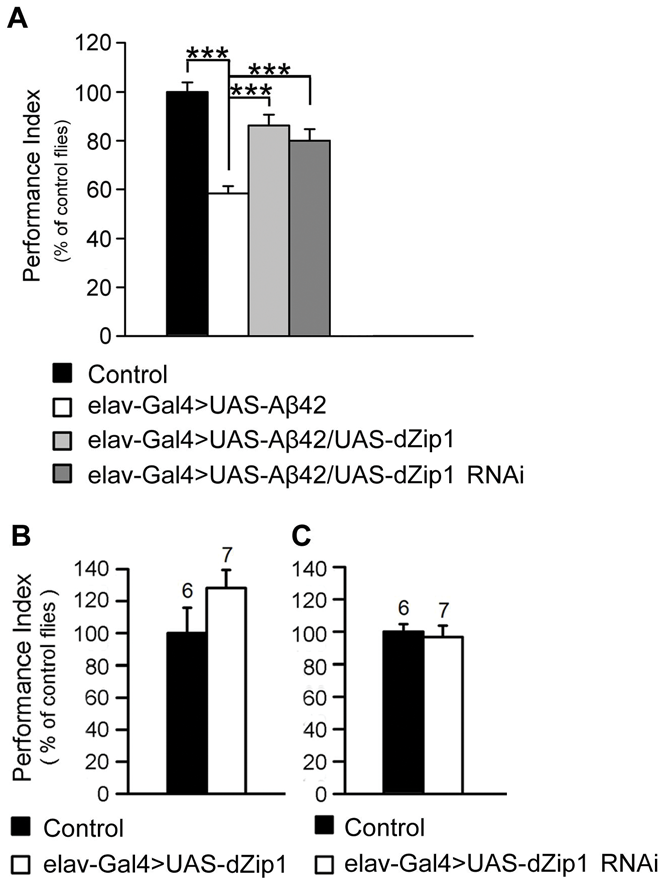
*dZip1* expression modulation ameliorates the Aβ42-induced immediate memory loss. (A) Expression of Aβ42 in fly brains induced an immediate memory loss compared with the control flies (*elav-Gal4>UAS-Aβ42 vs. UAS-Aβ42*) at 5-day-old. Both *dZip1* OE and RNAi in Aβ42-expressing fly brains (*elav-Gal4>UAS-Aβ42/UAS-dZip1* or *elav-Gal4>UAS-Aβ42/UAS-dZip1-RNAi 1#*) significantly ameliorated immediate memory loss. *t*-test, ***P<0.001. n = 8 PIs for each genotype. (B–C) Without expression of Aβ42, *dZip1* OE and RNAi had no effects on immediate memory (*elav-Gal4>UAS-dZip1* or *elav-Gal4>UAS-dZip1-RNAi 1# vs. elav-Gal4*), although *dZip1* OE mildly increased the memory score. n = 6–7 as indicated. All behavior data are normalized to the control flies. Data are expressed as means ± SEM.

A cardinal defect in Alzheimer's disease is memory loss. With extensively characterized Pavlovian olfactory aversive conditioning [29], a memory defect in adult Aβ42 flies started to appear as early as 5-day-old. *dZip1* knockdown significantly rescued memory loss at this stage (Figure 6A). Paradoxically, overexpression of *dZip1* also resulted in obvious memory recovery. As a control, we examined how alteration of *dZip1* expression alone (in the absence of Aβ42) might impact memory scores. Overexpressing or knocking down *dZip1* did not significantly influence memory of 5-day-old normal flies (Figure 6B and 6C), although overexpression of *dZip1* might have a marginal beneficial effect. These results suggest that modulating *dZip1* could alleviate the Aβ42 toxicity on memory ability at early stages.

### *dZip1* overexpression stimulates while *dZip1* inhibition decreases Aβ42 deposition

**Figure 7:**
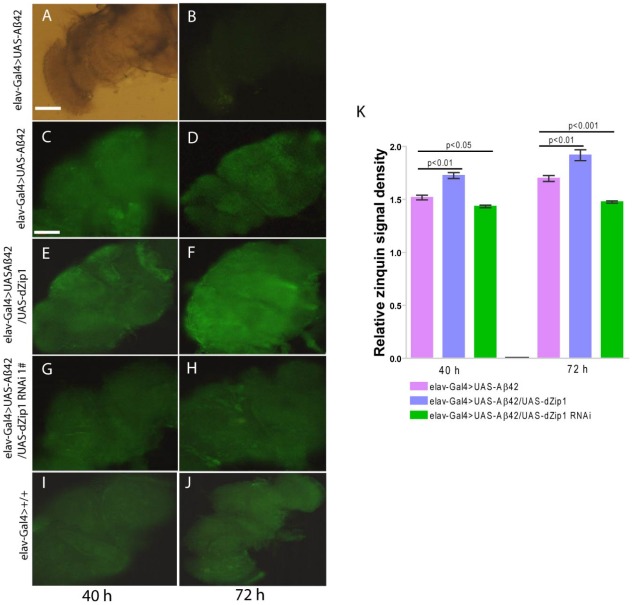
*dZip1* overexpression results in Zn accumulation in Aβ42-expressing fly brains revealed by Zinquin staining. 10-day old flies were treated with zinc-rich food (4 mM ZnCl_2)_ for 40 h and 72 h, respectively). Dissected brains were treated with 25 mM Zinquin for 30 min and then examined with a fluorescent microscope. No fluorescent signal was detected without Zinquin treatment (A–B). *dZip1 OE* (*elav-Gal4>UAS-Aβ42/UAS-dZip1*, E–F) led to an obvious Zn accumulation in the neocortex and neuropile region of the fly brains as compared with the control Aβ42 flies (*elav-Gal4>UAS-Aβ42*, C–D). Weaker signal was detected when *dZip1* was knocked down by RNAi (*elav-Gal4>UAS-Aβ42/UAS-dZip1-RNAi 1#*, G–H). *elav-Gal4* fly brains showed similar Zinquin signals as *dZip1-RNAi* fly brains (I–J). Relative Zinquin signals against background in the neocotex and neuropile region were quantitated using ImagJ software (K). The relative intensity of Zinquin signal, however, is by no menas a quantitative reflection of zinc levels [42]. Data are expressed as means ± SEM and analyzed by Student's *t*-test. n = 4 for each genotype. Scale bar, 100 µm.

**Figure 8:**
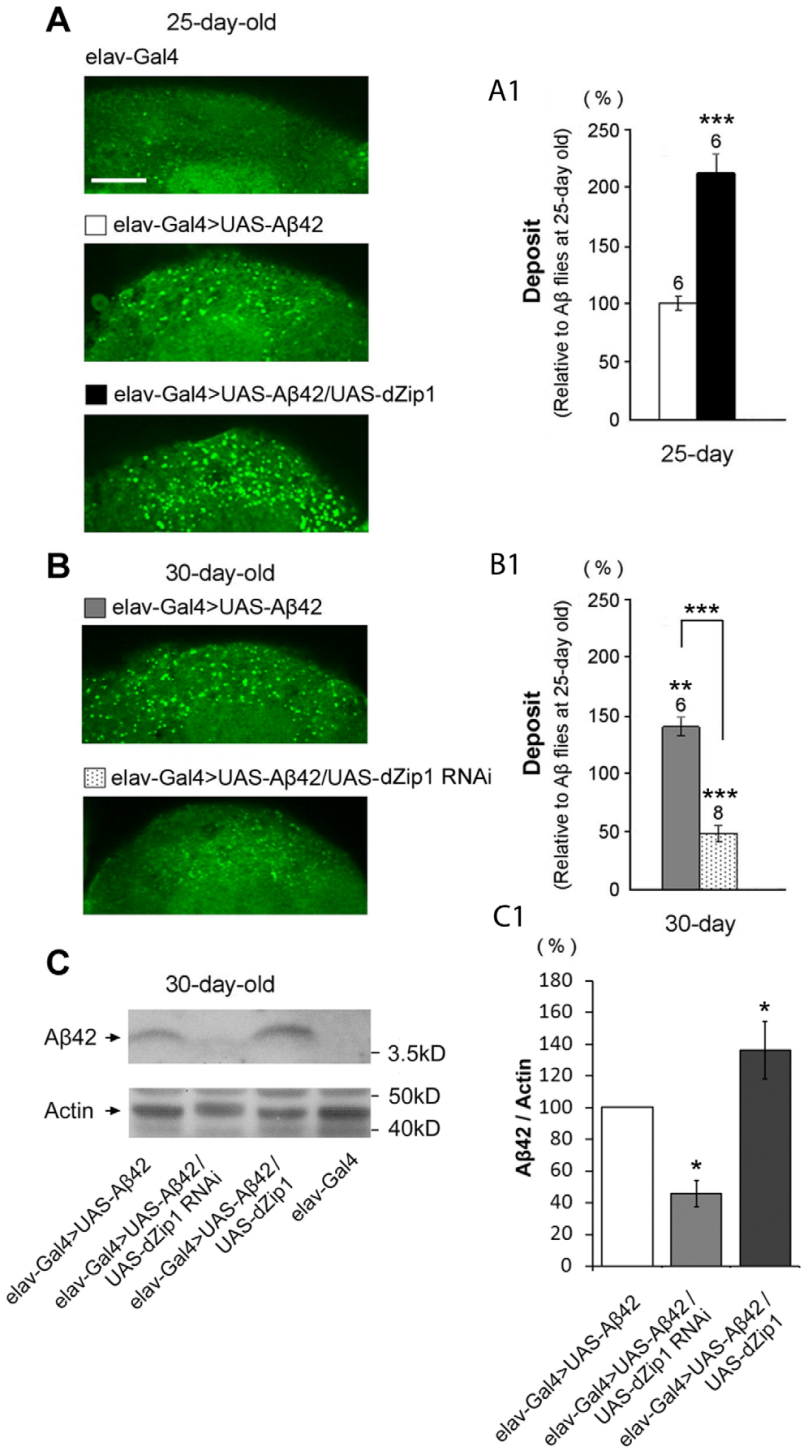
*dZip1* inhibition reduces the accumulation of Aβ42 fibril deposits as well as the SDS-soluble form. (A–B) Thioflavin-S (TS) staining was used to detect the Aβ42 fibril deposits in fly brains (bright green dots). Few deposits were found in control brains (*elav-Gal4*, A, top panel) at 25-day old. TS-positive deposits were found after Aβ42 expression in fly brains (*elav-Gal4>UAS-Aβ42*) at both 25 dae (A) and 30 dae (B). Quantitative contents of Aβ42 deposits based on TS signals were summarized and expressed after normalization to 25-day old Aβ42 flies (A1 and B1). The increase of Aβ42 deposits was age-dependent. Overexpressing *dZip1* in Aβ42-expressing brains (*elav-Gal4>UAS-Aβ42/UAS-dZip1*) significantly increased fibril deposits at 25 dae (A and A1). However, inhibiting *dZip1* (*elav-Gal4>UAS-Aβ42/UAS-dZip1-RNAi 1#*) dramatically decreased deposits density at 30 dae (B and B1). *t*-test, **P<0.01, ***P<0.001. Data are expressed as means ± SEM. n = 6 or 8 hemispheres for each genotype. Scale bar, 15 µm. (C) *dZip1* knockdown also decreases the low aggregated form of Aβ42. Protein lysates from 30-day old fly heads were prepared for western blotting assay. SDS-soluble Aβ42 were detected in *elav-Gal4>UAS-Aβ42* flies. Less and more SDS-soluble Aβ42 were found respectively when *dZip1* expression was inhibited and increased. (C1) is a quantitative measurement of (C). *t*-test, *P<0.05. n = 3 independent experiments. Data are expressed as means ± SEM.

The aforementioned experiments demonstrated dZip1 expression modulation can markedly alter the course of Aβ-associated neurodegeneration. Towards further analysis of the mechanism underlining dZip1 effect on Aβ toxicity, we first tried to determine where Zn was concentrated in these Aβ fly brains. We raised 10-day old Aβ42 flies on normal food supplemented with ZnCl_2_ and then used Zinquin staining to detect Zn distribution *in vivo*. The purpose of applying extra Zn is to enhance the fluorescent signal. Without Zinquin treatment, little signal was detected (Figure 7B). Over-expression of *dZip1* (Figure 7E and 7F) appeared to produce stronger signals in the neocortex and neuropile region of the fly brain compared with control Aβ42 (Figure 7C and 7D) flies. Quantitative analysis of these signal intensities showed a significant difference (Figure 7K, *t*-test, p<0.01). Only a faint signal was detected in *dZip1*-*RNAi* fly brains (Figure 7G, 7H and 7K, *t*-test, p<0.05 at 40 h and *t*-test, p<0.001 at 72 h). Staining of the brains of *elav-Gal4* flies without Aβ expression showed faint signals similar to *dZip1*-*RNAi* flies (Figure 7I and 7J). These results demonstrate a positive correlation between *dZip1* level and Zn accumulation in the neocortex and neuropile region of the fly brain where Aβ deposits were revealed (Figure 8).

To determine if Zinc is causally related to Aβ42 deposition, we overexpressed or knocked down *dZip1* under the control of *elav-Gal4* in Aβ42 flies and subjected the brains to histochemical analysis. Aβ42 peptides could form diffused amyloid deposits in fly brains [27], [28]. Thioflavin-S (TS) staining of the whole brain was used to specifically visualize the Aβ42 fibril deposits (Figure 8) [23]. Aβ42 deposits were observed in the Kenyon cell body region of Aβ42-expressing brains. The number of TS-positive deposits in Aβ42 flies was significantly increased with aging (Figure 8A and 8A1, 8B and 8B1). Over-expression of *dZip1* markedly increased the number of Aβ42 deposits compared with age-matched Aβ42 flies (Figure 8A and A1, ∼212% at 25-day old relative to Aβ42 flies at 25-day old, *t*-test, p<0.001). Conversely, RNAi-based knockdown of *dZip1* in Aβ42 flies significantly decreased the Aβ42 deposits compared with age-matched Aβ42 flies (Figure 8B and B1, ∼48% at 30-day old relative to Aβ42 flies at 25-day old, *t*-test, p<0.001). Taken together, our results demonstrate that dZip1 over-expression can increase Aβ42 accumulation whereas inhibiting dZip1 can decrease Aβ42 deposition.

### Modulation of dZip1 expression also affects the low aggregation form of Aβ

The above TS staining was used to specifically detect the Aβ42 fibril deposits. Recently, an alternative model for the Aβ toxicity is put forth hypothesizing that amyloid oligomers rather than plaques are responsible for the disease [30]. The oligomer form, together with the monomeric form of Aβ, is soluble in SDS whereas the fibril aggregate is not. We next investigated how SDS-soluble Aβ was affected by modulating dZip1 expression. Fly brain lysates were used for a Western blotting analysis. The result showed that the SDS-soluble Aβ42 (low level aggregation forms) was dramatically decreased in accordance to reduced dZip1 level and increased when dZip1 was over-expressed (Figure 8C and 8C1). In a separate experiment, SDS-insoluble but formic acid-soluble Aβ42 (high level aggregation forms) was also examined; much decreased formic acid-soluble Aβ42 level was observed when dZip1 expression was inhibited (Figure S4), consistent with the TS-staining result.

**Figure 9:**
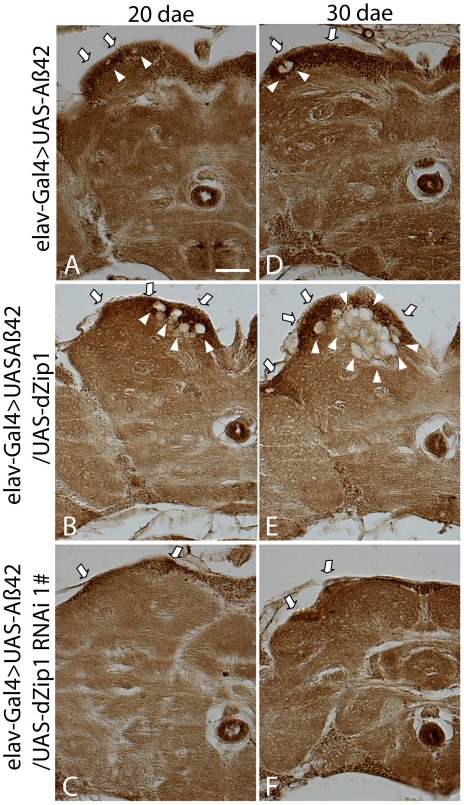
Forcible dZip1 expression modulation could alter Aβ42 accumulation in the brain. Paraffin sections of 20- or 30-day old fly brains were stained with antibody against Aβ42. Aβ42 deposits were primarily accumulated in the neuronal somatic region (arrow). (B and E) shows *dZip1* OE produced more Aβ42 deposits than the age-matched control (A and D), while *dZip1* RNAi reduced Aβ42 deposits (C and F). Scale bar, 50 µm.

Whole-mount immunohistochemical staining with Aβ42 antibody also revealed a reduction of Aβ42 level in the *dZip1*-*RNAi* fly brains (Figure 9). Abundant amyloid deposits were observed in the Kenyon cell body region of 20-day old Aβ42-expressing brains (Figure 9A, arrow). Such deposits were markedly decreased when *dZip1* was knocked down (Figure 9C) and greatly increased when *dZip1* was over-expressed (Figure 9B). Similar results were found in 30-day old fly brains (Figure 9E and 9F) compared with age-matched Aβ42-expressing control brains (Figure 9D). In the case of co-overexpression of dZip1 and Aβ42, vacuolization became much more pronounced with aging (Figure 9E). As a control for the Aβ42 antibody, we used it against *elav-Gal4* fly brains and found no meaningful signal, indicating the antibody reacts specifically with Aβ (Figure S5).

**Figure 10:**
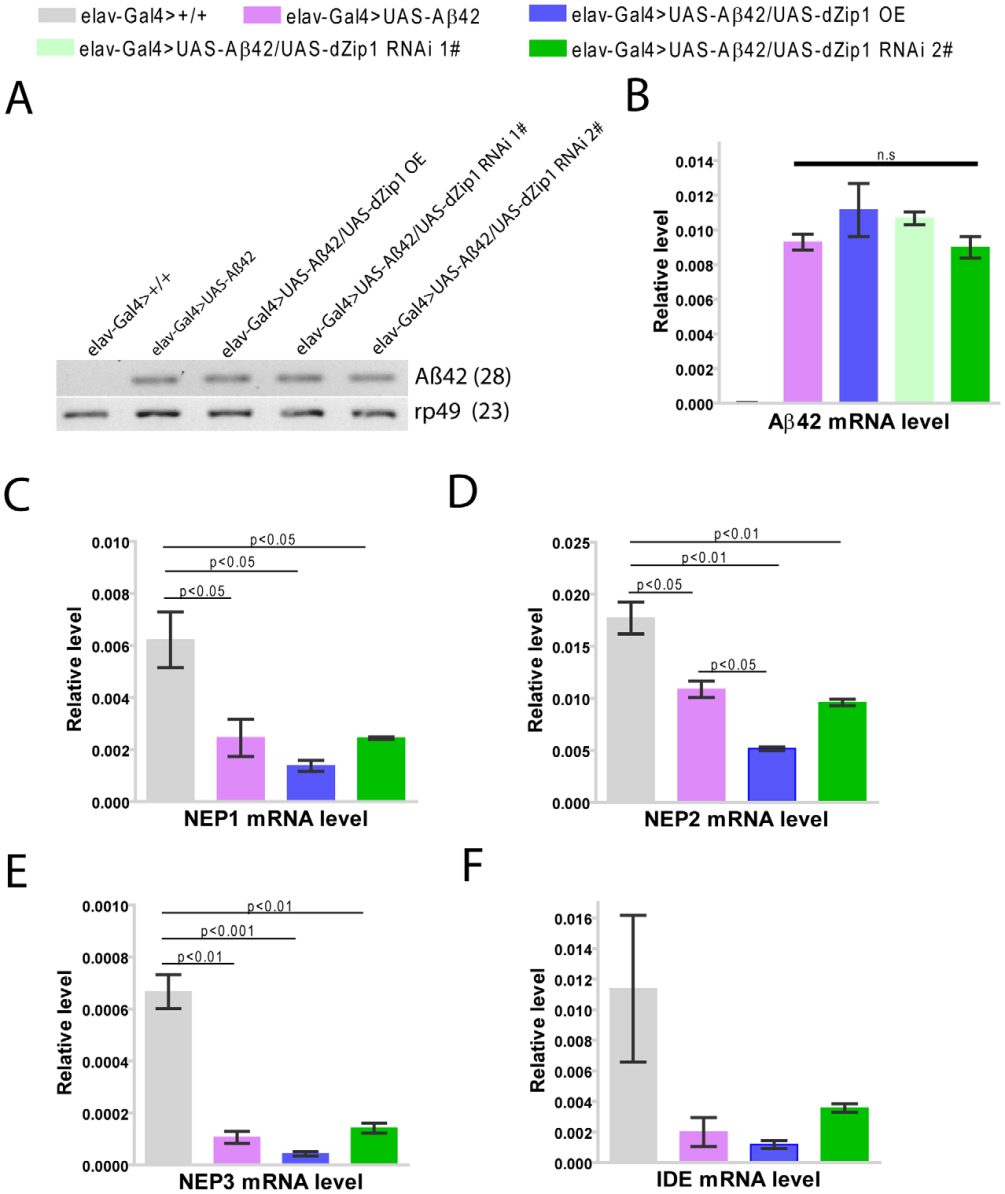
The rescuing effect of *dZip1* RNAi on Aβ appears not mediated by affecting Aβ42 nor NEP or IDE expression in the Aβ42 flies. (A) shows a representative of sqRT-PCR analysis of Aβ42 expression in brains of 20-day-old flies. No Aβ42 expression was detected in brains of *elav-Gal4>+/+*flies. *dZip1* RNAi or OE did not significantly influence the expression level of Aβ42 compared to control *elav-Gal4>UAS-Aβ42* flies. *rp49* was used as the loading control. (B) Aβ42 expression in brains of 20-day-old flies was determined by qRT-PCR. Statistic analysis showed no significant differences among *elav-Gal4>UAS-Aβ42*, *elav-Gal4>UAS-Aβ42/UAS-dZip1OE*, *elav-Gal4>UAS-Aβ42/UAS-dZip1 RNAi 1#*, and *elav-Gal4>UAS-Aβ42*/*UAS-dZip1 RNAi 2#*. *t*-test, P>0.05 (in comparison with *elav-Gal4>UAS-Aβ42* flies). n = 3 biological repeats. (C)–(F) shows *Drosophila NEP1*, *NEP2*, *NEP3* and *IDE* expression in brains of 30 day old flies. All the four degrading enzymes shows higher expression level in fly brains without Aβ42 expression than that with Aβ42 expression. *dZip1* RNAi did not significantly influence NEP or IDE expression in Aβ42 flies compared to the control Aβ42 flies, while *dZip1* OE shows a trend of reducing NEP or IDE expression level, but only statistically significant for NEP2. The relative Aβ42, NEP1-3 and IDE expression levels against *rp49* were from three independent biological replicates and plotted with SEM (error bars).

Because *dZip1* RNAi led to a general reduction of Aβ42 level, we tried to ask whether this was due to Aβ42 expression inhibition or an increase of Aβ42 degradation. Aβ42 gene was directly under the control of elav-Gal4, and indeed no changes of RNA expression under the various genetic manipulations were observed (Figure 10A and 10B, Figure S2B). We then suspected that zinc might reduce the rate of Aβ42 clearance. Several proteases (*NEP1-3*, *IDE*) were proposed to act in the Aβ degradation [31], [32] and we thus explored whether they were affected by dZip1 expression in the Aβ flies. Aβ expression brought some changes to the expression of these genes, but introduction of *dZip1-RNAi* transgene in the Aβ flies resulted no significant expression increase of these genes (Figure 10C-10F). We did however, observed some decrease of *NEP2* expression in dZip1 OE/Aβ flies. Therefore we saw little evidence that the observed rescuing effect of *dZip1* RNAi on Aβ42 flies is mediated by an increase of these degrading proteases.

Together, we conclude *dZip1* reduction decreases levels of Aβ42, in both the high (fibril aggregates) and low aggregation forms.

## Discussion

Previous studies suggest that heavy metals, especially Zn, have a close relationship with the development of Alzheimer's disease [4]–[6], [8]–[10]. However, little genetic evidence exists that demonstrates a functional link between Aβ and proteins involved in zinc assimilation. In this study, we showed that manipulating the *Slc39* family protein dZip1 greatly altered the Aβ toxicity. In particular, knocking down *dZip1* in brains of Aβ42 flies markedly decreased both Aβ42 deposits and zinc accumulation. *dZip1* knockdown ameliorated early memory loss, decreased the number of neurodegenerative vacuoles, significantly enhanced locomotor ability and prolonged life-span in Aβ42-expressing flies. Taken together, our results provide strong evidence to support our hypothesis that knocking down protein dZip1 may mitigate Aβ pathology and Aβ-dependent behavioral defects in a *Drosophila* model of Alzheimer's disease.

The accumulation and aggregation of Aβ42 peptide in the neocortex has been suggested to be caused by its abnormal interactions with neocortical metal ions especially Zn, which is constitutively found at high levels in the neocortical regions where they play important roles in normal physiology [11], [33], [34]. Our qRT-PCR results showed that the *dZip1* transcript level was higher in brains of young control *elav-Gal4* and *w^1118^* flies and decreased with age, but lower in brains of young Aβ42 flies and increased with age. Paradoxically, *dZip1* transcript level was lower in young adult Aβ42 flies than age matched normal flies. Since at this stage Zn level is not any lower (Figure 2B) in Aβ42 flies, we suspect other Zn homeostasis genes might also be affected. Indeed, besides *dZip1* quite a few other Zip or ZnT (*Slc*30 family transporter) genes are also expressed in the brain (data not shown and the Flyatalas: http://flyatlas.org), likely contributing to Zn uptake or export. Supporting this notion is a recent report showing that the expression level of the *Slc*30 family protein ZnT3 decreased with age in AD brains [35], [36]. Thus the general Zn status is the result from the combination effect of all these Zn homeostasis genes. It is possible that Aβ42 expression alters the Zn homeostasis starting from an early stage, although we are not totally clear why *dZip1* is reduced at early stages but increased at late stages. One thing worthy of consideration is that total zinc level does not reflect well available cellular zinc. In other words, two brains with the same level of total zinc may have very different levels of zinc for use. dZip1 expression regulation may reflect the cell's native response to its own physiological states-to bring more or less zinc into cells. Because Aβ42 can likely bind to zinc, and monomer and oligomers may have different binding characteristics, we speculate that Aβ42 can affect zinc homeostasis even in the absence of noticeable total zinc level alteration. This dyshomeostasis could result or be reflected by native dZip1 expression changes.

Not all pathogenic effects of Aβ in *Drosophila* correlate directly with its aggregation. An artificial mutation (L17P) with decreased Aβ42 aggregation tendency is associated with lower toxicities, in term of locomotor ability and lifespan, but induces even earlier onset of memory defects than its normal counterpart [37]. Furthermore, although both Aβ40 and Aβ42 affect learning, only Aβ42 causes degeneration [27]; inhibition of PI3K activity ameliorated the Aβ42-induced early memory loss, but did not rescue neurodegeneration [38]. These results lead to the speculation that neuronal dysfunction and neurodegeneration may be mediated by different mechanisms. In our study, while knocking down *dZip1* and overexpressing *dZip1* were associated with opposite effects on all other aspects of Aβ42-induced toxicity, it is interesting that both knocking down *dZip1* or overexpressing *dZip1* lessened Aβ42-induced early memory loss. While inhibiting Zn accumulation in fly brains could promote memory in Aβ42-expressing flies by reducing aggregation of Aβ42, the amelioration of memory loss with dZip1-overexpression is likely due to a different mechanism. At the moment, we are not certain how this memory gain with *dZip1* over-expression was achieved.

One might ask whether the dramatic effect seen with *dZip1* modulation in Aβ42 flies could be reproduced with dietary zinc supplement or chelation. High levels of zinc supplement (such as 2.5 mM) do significantly worsen the viability of Aβ42 flies, however at this level normal flies are also affected (Figure S3C and S3D). Using a zinc chelator clioquiniol, dietary feeding can rescue to some extent Abeta flies, interestingly mostly in male flies (Figure S3A and S3B). Thus genetic intervention is a much more effective method. We interpret this as systemic Zn overloading may cause damages to other tissues before enabling a dramatic Zn increase in the target organ. Likewise, zinc depletion at the organismal level is generally harmful: zinc is an essential nutrient vital to many biological processes. Indeed, high levels of clioquinol greatly impact even the survival of normal flies. Thus, we believe low zinc level can affect fly development and survival so that overall beneficial effect of zinc reduction by dietary measures is significantly less effective than targeted neuronal zinc reduction through genetic interventions.

**Figure 11:**
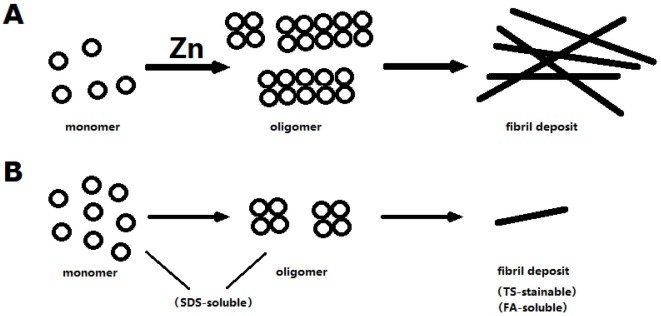
A model to explain zinc's effect on Aβ. Zinc stimulates the polymerization of Aβ, resulting more oligomer formation, which in turn produces more fibril deposit. When zinc is low, less oliogomers are formed. The monomers may be more susceptible to degradation than the polymers/fibrils so that the observed overall Aβ level is reduced when zinc is low.

dZip1 repression results in less Aβ42 level. Although other possibility cannot be excluded, we favor the model that zinc induces oligomerization of Aβ42, as supported by numerous *in vitro* evidences. More oligomers result more fibril deposits. Perhaps the oligomer and the aggregated Aβ42 are more stable than the monomer, so that the Aβ42 level is dramatically reduced in *dZip1* RNAi flies, where a larger fraction of Aβ42 adopt the monomeric form more susceptible to clearance (Figure 11).

In summary, we have demonstrated the modulating effect of *dZip1* on Aβ42 toxicity in a *Drosophila* model of Alzheimer's disease. We observed Aβ42 expression could cause a change of *dZip1* expression pattern during ageing. Through genetic manipulation of *dZip1* expression, we can modify the pathological process of Aβ42. These results raise the possibility that *Zip1*, or more broadly Zn transporter genes expressed in the brain, could be a new kind of promising therapeutic target in AD pathology.

## Materials and Methods

### *Drosophila* genetics and DNA constructs

Flies were raised and maintained at 25°C or otherwise indicated temperatures. All general stocks were obtained from the Bloomington *Drosophila* Stock Center, which includes *Actin-Gal4*, *elav-Gal4*. *UAS-Aβ42* transgenic strain was reported previously [37]. To make *UAS-dZip1* transgenic fly, corresponding genomic DNA including a 76 bp intron was cloned into the *pUAST* vector. The primers used for PCR amplification were: UAS-dZip1-F: 5′-CCGAATTCAAGATGAGCGCTACCGC-3′ and UAS-dZip1-R: 5′- GGAAGATCTCTA GGAACAGGTTAGGCTG-3′. The *UAS-dZip11-RNAi* constructs were generated according to Lee and Carthew [39]. The primers used were: WIZ-dZip1-RNAi-F: 5′-GGGTCTAGAATGAGCGCTACCGC-3′ and WIZ-dZip1-RNAi-R: 5′-GGTCTAGACC ACACAGTGCTCACAG-3′. All transgenic flies were generated in *w^1118^* background following standard protocols.

### RNA isolation, semi-quantitative and quantitative RT–PCR

Total RNA was extracted from the brain, gut and carcase (whole body minus brain and gut) of 10 adults for each sample using TRIzol Reagent (Invitrogen) according to the manufacturers' instructions and subjected to DNA digestion using DNAse I (Ambion) immediately. The concentration and quality of DNAse-treated total RNA were then tested, and 800 ng total RNA from each sample was used to synthesize cDNA by using Superscript™ II Reverse Transcriptase kit (Invitrogen) with oligo(dT) primers. Semi-quantitative RT-PCR (sqRT-PCR) was performed using primers for *rp49* (forward: 5′- TACAGGCCCAAGATCGTGAA-3′; reverse: 5′- TCTCCTTGCGCTTCTTGGA-3′) and *dZip1* (forward: 5′-ATTATCCTCGCCCTTTCGC-3′; reverse: 5′-TCACCCTCCGCT TCGTCAG-3′). *rp49* was used as the loading control. For quantitative RT-PCR (qRT-PCR), 20 fly brains were used for each sample, RNA extraction and cDNA synthesis were the same as described for sqRT-PCR. Primers for amplifying *dZip1*, NEP1, NEP2, NEP3 and Aβ42 were listed as Table S1. Real-time PCR reactions were monitored on an iCycler (Bio-Rad) by means of SYBR Green (Bio-Rad) dye. mRNA expression levels were determined relative to *rp49* expression by relative quantification. Statistical analysis was performed using the Student's *t-*tests.

### Histology and immunostaining

For immunostaining analysis on paraffin sections, antigen retrieval was achieved by boiling the samples in 10 mM sodium citrate (pH 6.6) for 15 min. Immunostaining was performed using an avidin-biotin-peroxidase complex (ABC) kit (Vector Laboratories). For Aβ staining, the primary antibody used was anti-Aβ42 (Promega; 1∶500). Appropriate secondary antibodies were diluted 1∶200, and histochemical detection was done with DAB (Sigma-Aldrich) color development.

### Quantification of neurodegeneration

Adult fly heads were fixed in Carnoy solution (ethanol∶chloroform∶acetic acid = 6∶3∶1) overnight at 4 C°, and then embedded in the paraffin and sectioned at 6 m thickness. H&E staining was performed following standard protocols. Neurodegeneration was assessed by quantification of vacuoles with diameter greater than 3 µm in the fly brains. At least five fly brains were analyzed for each genotype.

### Fibril Aβ42 deposit detection

Thioflavin-S (TS, Sigma) staining was performed to detect fibril Aβ42 deposits. Fly brains were fixed in 4% paraformaldehyde and permeabilized by 2% triton. Brains were then transferred to 0.25% TS in 50% ethanol overnight. After 1× wash for 10 min in 50% ethanol and 3× wash with PBS, they were mounted with focusclear (Pacgen Biopharmaceuticals Inc.) and covered by cover slips. Slides were inspected with a Zeiss LSM 510 confocal microscope, aided by LSM 510 analysis software. TS-positive deposits located in the mushroom body somatic region were counted for comparison analysis.

### Pavlovian olfactory associative memory recording

The training and testing procedures were as previously described [40], [41]. During one training session, a group of 100 flies was sequentially exposed for 60 s to two odors, octanol (OCT) or methylcyclohexanol (MCH), with 45 s of fresh air in between. Flies were subjected to foot-shock (1.5 s pulses with 3.5 s intervals, 60 V) during exposure to the first odor (CS+) but not to the second (CS−). To measure “immediate memory (also referred to as “learning”)”, flies were transferred immediately after training to the choice point of a T-maze and forced to choose between the two odors for 2 min, at which time they were trapped in their respective T-maze arms, anesthetized, and counted. A performance index (PI) was calculated from the distribution of flies in the T-maze. A reciprocal group of flies was trained and tested by using OCT as the CS+ and MCH as the CS+, respectively. PIs from these two groups finally were averaged for an *n* = 1 and multiplied by 100. A PI of 0 represented a 50∶50 distribution, whereas a PI of 100 represented 100% avoidance of the shock-paired odor.

### Western blot analysis

SDS-soluble and SDS-insoluble but formic acid-soluble Aβ42 were prepared as previously reported [27]. Lysates from equal number of fly heads were diluted in SDS sample buffer and separated by 10–20% Tris-Tricine gels (Invitrogen), and transferred to nitrocellulose membranes (Invitrogen). Membranes were boiled in PBS for 3 min. Membranes were blocked with 3% BSA and blotted with primary antibody. Primary antibodies used in this study were mouse anti-Aβ42 (6E10, Covance Research Products) and rabbit anti-Actin (Sigma). After washing in TBST for 3 times, membranes were incubated with secondary antibodies for 1 hr at RT. After 3 times wash in TBST, membranes were incubated with ECL working solution (GE healthcare) and developed with films (Kodak). Data were analyzed with ImageJ sofeware (NIH).

### Zinquin staining

10-day old adult flies reared on normal condition were transferred to vials with normal food supplied with 4 mM ZnCl_2_. Fly brains were then dissected at 40 and 72 h after transferring, respectively. The dissected brains were then incubated with 25 µM Zinquin (Sigma) for 30 min at 37°C, and washed 3 times with 1×PBS buffer for 5 min each time. After that, brains were examined using conventional epifluorescence microscope (Nikon, Diaphot 300) equipped with a Nikon 100×, 1.4 NA Plan Apo oil-immersion objective. Zinquin signals in the neocortex and neuropile region of fly brains were quantitated by using ImagJ software.

### Metal stress and content assay

For the metal stress assay, *Drosophila* was fed on normal medium containing 2.5 mM ZnSO_4_. Control flies were fed on normal medium in the absence of drug. Mortality was recorded every 24 h or a longer intervals. Each vial contained 20–25 flies, and the experiments were repeated at least three times.

For the metal content analysis, flies were reared on normal food and fly heads were collected at day 7, 20 and 30 after eclosion. Fly heads were dissolved in 1 ml 65% HNO_3_, boiled in 100°C water bath for 10 min and diluted to 10 ml for metal content analysis with inductively coupled plasma optical emission spectrometry (ICP-OES, IRIS Intrepid II XSP, Thermo Electron Corporation, USA).

### Climbing assay

The climbing assay was referenced to Iijima et al. (2004) [27]. Briefly, twenty flies were placed in a plastic vial and gently tapped to the bottom. The number of flies at the top of the vial was counted after 18 s of climbing under red light (Kodak, GBX-2, Safelight Filter). The data shown represent results from a cohort of flies with four repeats tested serially for 5–50 days. The experiment was repeated more than three times.

### Longevity assay

Flies of two days after eclosion were used for the experiment. Twenty to 23 flies were placed in a food vial. Each vial was kept at 25 or 29°C, 70% humidity, under a 12-h light–dark cycle. Food vials were changed every 2–3 days, and dead flies were counted at that time. At least 150 flies were prepared for each genotype, and the experiments were carried out more than three times. Percent increases in life span are based on comparing the median survivals. Prism (GraphPad) was used for statistical analysis of lifespan data. Mantel-Cox log-rank statistical analysis was used for testing statistical significance of the differences between the survivorship curves.

### Statistical analysis

All data were analyzed by Student's *t*-test. Statistical results were presented as means ± SEM. Asterisks indicate critical levels of significance (*P<0.05, **P<0.01 and ***P<0.001).

## Supporting Information

Figure S1 20-day old *Drosophila* brain zinc levels are affected by Aβ42 and dZip1 expression. Zn content in brains of 20-day old flies was measured by ICP-OES. *dZip1* OE (*elav-Gal4>UAS-Aβ42/UAS-dZip1*) facilitated Zn accumulation in fly brains compared with control Aβ42 flies (*elav-Gal4 vs. elav-Gal4>UAS-Aβ42*). *dZip1* RNAi slowed down the brain Zn accumulation process (*elav-Gal4>UAS-Aβ42/UAS-dZip1-RNAi 2#* and *elav-Gal4>UAS-Aβ42/UAS-dZip1(v3986) RNAi*). Data on top of the bars represent the relative increased percentage of Zn level to control *elav-Gal4>+/+* flies. Data are expressed as means ± SEM and analyzed by Student's *t*-test. n = 3 for each genotype.
(TIF)

Figure S2 V3986, an independent dZip1 RNAi line, could similarly rescue Aβ-associated brain vacuolization and climbing defect. (A) shows a representive of sqRT-PCR analysis of dZip1 expression in brains of 5-day old flies.V3986 RNAi line shows similar knock down effect with dZip1 RNAi 2#, which is better than v3987 RNAi line. *rp49* was used as the loading control. (B) shows a representive of sqRT-PCR analysis of Aβ42 expression in brains of 30-day old flies.V3986 RNAi line shows similar Aβ42 expression level with dZip1 RNAi 2# line and dZip1 OE line, and no reducing Aβ42 expression level was found compared to Aβ42 flies. *rp49* was used as the loading control. (C) Paraffin sections of 30-day old fly brains were stained with H&E. V3986 RNAi line produced similar rescuing effect as dZip1 RNAi 2# line on Aβ42 expression induced neurodegeneration (arrowheads indicate the vacuoles). Scale bar, 50 µm. (D) is a statistical analysis of (C) Aβ42-induced neurodegeneration under *dZip1* expression modulation. Number of vacuoles (diameter>3 µm) on each section was counted and summarized. V3986 RNAi line produce similar rescuing effect as dZip1 RNAi 2# line, which has significant less vacuole numbers than control *elav-Gal4>UAS-Aβ42* flies (p<0.001). Data are expressed as means ± SEM and analyzed by the Student's *t*-test. n = 9 for each genotype. (E) V3986 RNAi line has similar rescuing effect as dZip1 RNAi 2# line on Aβ42 expression induced climbing defect. *t*-test, **P<0.01, ***P<0.001 (in comparison with *elav-Gal4>UAS-Aβ42* flies). n = 6 independent experiments.
(TIF)

Figure S3 Zinc chelation with clioquinol extends while zinc addition shortens Aβ42 survival. Male (A) and female (B) *elav-Gal4>UAS-Aβ42* flies were raised on normal food with DMSO (control) and normal food supplied with 0.5 mM clioquinol (CQ) at 29°C. (C) shows the survival curves of *elav-Gal4* and *elav-Gal4>UAS-Aβ42* flies raised on normal food or supplied with 2.5 mM ZnSO_4_, at 25°C. (D) was a Mantel-Cox log-rank statistical analysis of (C) survival curves. The percentage of survivorship was plotted against the age (dae). Foods were changed every 2–3 days, and the survival numbers of flies were counted. At least three biological repeats were used for each genotype.
(TIF)

Figure S4 *dZip1* expression reduction decreases both the low and high aggregated forms of Aβ42. Protein lysates from equal number of 30-day old fly heads were prepared for western blotting assay. (A) shows a representative of western blot experiments. No Aβ42 was detected in control *elav-Gal4* flies. SDS-soluble and SDS-insoluble but formic acid-soluble Aβ42 were detected in *elav-Gal4>UAS-Aβ42* flies. Less SDS-soluble Aβ42 and no formic acid-soluble Aβ42 were found when *dZip1* expression was inhibited by RNAi. (B) Statistical analysis of SDS soluble Aβ42 bands in (A). *t*-test, **P<0.01 (in comparison with *elav-Gal4>UAS-Aβ42* flies). n = 3 independent experiments.
(TIF)

Figure S5 The Aβ42 antibody is specific to Aβ42 in the fly brain. Paraffin sections of 20-day old *elav-Gal4* and *elav-Gal4>UAS-Aβ42* fly brains were stained with antibody against Aβ42. Pan-neuronal expression of Aβ42 in fly brains induced Aβ42 accumulation which was primarily located in the neuronal somatic region (arrow). No Aβ42 signals were detected in age-matched control brains without Aβ42 expression. Scale bar, 50 µm.
(TIF)

Table S1 Real-time RT–PCR primers used in this study.
(DOC)

## References

[1] Hardy J, Selkoe DJ (2002). Medicine - The amyloid hypothesis of Alzheimer's disease: Progress and problems on the road to therapeutics.. Science.

[2] Hardy J (2006). A hundred years of Alzheimer's disease research.. Neuron.

[3] Walsh DM, Selkoe DJ (2007). Abeta Oligomers - a decade of discovery.. Journal of Neurochemistry.

[4] Danscher G, Jensen KB, Frederickson CJ, Kemp K, Andreasen A (1997). Increased amount of zinc in the hippocampus and amygdala of Alzheimer's diseased brains: A proton-induced X-ray emission spectroscopic analysis of cryostat sections from autopsy material.. Journal of Neuroscience Methods.

[5] Lovell MA, Robertson JD, Teesdale WJ, Campbell JL, Markesbery WR (1998). Copper, iron and zinc in Alzheimer's disease senile plaques.. Journal of the Neurological Sciences.

[6] Frederickson CJ, Bush AI (2001). Synaptically released zinc: Physiological functions and pathological effects.. Biometals.

[7] Atwood CS, Moir RD, Huang XD, Scarpa RC, Bacarra NME (1998). Dramatic aggregation of Alzheimer Abeta by Cu(II) is induced by conditions representing physiological acidosis.. Journal of Biological Chemistry.

[8] Bush AI, Pettingell WH, Multhaup G, Paradis MD, Vonsattel JP (1994). Rapid Induction of Alzheimer a-Beta Amyloid Formation by Zinc.. Science.

[9] Frederickson CJ, Koh JY, Bush AI (2005). The neurobiology of zinc in health and disease.. Nature Reviews Neuroscience.

[10] Stoltenberg M, Bush AI, Bach G, Smidt K, Larsen A (2007). Amyloid plaques arise from zinc-enriched cortical layers in APP/PS1 transgenic mice and are paradoxically enlarged with dietary zinc deficiency.. Neuroscience.

[11] Opazo C, Huang XD, Cherny RA, Moir RD, Roher AE (2002). Metalloenzyme-like activity of Alzheimer's disease beta-amyloid. Cu-dependent catalytic conversion of dopamine, cholesterol, and biological reducing agents to neurotoxic H2O2.. Journal of Biological Chemistry.

[12] Samudralwar DL, Diprete CC, Ni BF, Ehmann WD, Markesbery WR (1995). Elemental Imbalances in the Olfactory Pathway in Alzheimers-Disease.. Journal of the Neurological Sciences.

[13] Deibel MA, Ehmann WD, Markesbery WR (1996). Copper, iron, and zinc imbalances in severely degenerated brain regions in Alzheimer's disease: Possible relation to oxidative stress.. Journal of the Neurological Sciences.

[14] Cornett CR, Markesbery WR, Ehmann WD (1998). Imbalances of trace elements related to oxidative damage in Alzheimer's disease brain.. Neurotoxicology.

[15] Mantyh PW, Ghilardi JR, Rogers S, Demaster E, Allen CJ (1993). Aluminum, Iron, and Zinc Ions Promote Aggregation of Physiological Concentrations of Beta-Amyloid Peptide.. Journal of Neurochemistry.

[16] Bush AI, Moir RD, Rosenkranz KM, Tanzi RE (1995). Zinc and Alzheimers-Disease - Response.. Science.

[17] Clements A, Allsop D, Walsh DM, Williams CH (1996). Aggregation and metal-binding properties of mutant forms of the amyloid Abeta peptide of Alzheimer's disease.. Journal of Neurochemistry.

[18] Esler WP, Stimson ER, Jennings JM, Ghilardi JR, Mantyh PW (1996). Zinc-induced aggregation of human and rat beta-amyloid peptides in vitro.. Journal of Neurochemistry.

[19] Cherny RA, Atwood CS, Xilinas ME, Gray DN, Jones WD (2001). Treatment with a copper-zinc chelator markedly and rapidly inhibits beta-amyloid accumulation in Alzheimer's disease transgenic mice.. Neuron.

[20] White AR, Du T, Laughton KM, Volitakis I, Sharples RA (2006). Degradation of the Alzheimer disease amyloid beta-peptide by metal-dependent up-regulation of metalloprotease activity.. Journal of Biological Chemistry.

[21] Donnelly PS, Caragounis A, Du T, Laughton KM, Volitakis I (2008). Selective intracellular release of copper and zinc ions from bis(thiosemicarbazonato) complexes reduces levels of Alzheimer disease amyloid-beta peptide.. Journal of Biological Chemistry.

[22] Li C, Wang J, Zhou B (2010). The metal chelating and chaperoning effects of clioquinol: insights from yeast studies.. Journal of Alzheimer's Disease.

[23] Cousins RJ, Liuzzi JP, Lichten LA (2006). Mammalian zinc transport, trafficking, and signals.. Journal of Biological Chemistry.

[24] Lichten LA, Cousins RJ (2009). Mammalian Zinc Transporters: Nutritional and Physiologic Regulation.. Annual Review of Nutrition.

[25] Wang XX, Zhou B (2010). Dietary Zinc Absorption: A Play of Zips and ZnTs in the Gut.. Iubmb Life.

[26] Huang LP, Kirschke CP, Zhang YF, Yu YY (2005). The ZIP7 gene (Slc39a7) encodes a zinc transporter involved in zinc homeostasis of the Golgi apparatus.. Journal of Biological Chemistry.

[27] Iijima K, Liu HP, Chiang AS, Hearn SA, Konsolaki M (2004). Dissecting the pathological effects of human Abeta40 and Abeta42 in *Drosophila*: A potential model for Alzheimer's disease.. Proceedings of the National Academy of Sciences of the United States of America.

[28] Finelli A, Kelkar A, Song HJ, Yang HD, Konsolaki M (2004). A model for studying Alzheimer's Abeta 42-induced toxicity in *Drosophila* melanogaster.. Molecular and Cellular Neuroscience.

[29] Tully T, Quinn WG (1985). Classical-Conditioning and Retention in Normal and Mutant *Drosophila-Melanogaster*.. Journal of Comparative Physiology A: Sensory Neural and Behavioral Physiology.

[30] Walsh DM, Selkoe DJ (2007). Aβ oligomers—a decade of discovery.. J Neurochem.

[31] Farris W, Mansourian S, Chang Y, Lindsley L, Eckman EA (2003). Insulin-degrading enzyme regulates the levels of insulin, amyloid b-protein, and the b-amyloid precursor protein intracellular domain in vivo.. Proc Natl Acad Sci USA.

[32] Iwata N, Tsubuki S, Takaki Y, Watanabe K, Sekiguchi M (2003). Identification of the major Abeta1-42-degrading catabolic pathway in brain parenchyma: suppression leads to biochemical and pathological deposition.. Nat Med.

[33] Frederickson CJ, Suh SW, Silva D, Frederickson CJ, Thompson RB (2000). Importance of zinc in the central nervous system: The zinc-containing neuron.. Journal of Nutrition.

[34] Suh SW, Jensen KB, Jensen MS, Silva DS, Kesslak PJ (2000). Histochemically-reactive zinc in amyloid plaques, angiopathy, and degenerating neurons of Alzheimer's diseased brains.. Brain Research.

[35] Beyer N, Coulson DTR, Heggarty S, Ravid R, Irvine GB (2009). ZnT3 mRNA levels are reduced in Alzheimer's disease post-mortem brain.. Molecular Neurodegeneration.

[36] Adlard PA, Parncutt JM, Finkelstein DI, Bush AI (2010). Cognitive Loss in Zinc Transporter-3 Knock-Out Mice: A Phenocopy for the Synaptic and Memory Deficits of Alzheimer's Disease?. Journal of Neuroscience.

[37] Iijima K, Chiang HC, Hearn SA, Hakker I, Gatt A (2008). Abeta 42 Mutants with Different Aggregation Profiles Induce Distinct Pathologies in *Drosophila*.. PLoS ONE.

[38] Chiang HC, Wang L, Xie ZL, Yau A, Zhong Y (2010). PI3 kinase signaling is involved in Abeta-induced memory loss in *Drosophila*.. Proceedings of the National Academy of Sciences of the United States of America.

[39] Lee YS, Carthew RW (2003). Making a better RNAi vector for *Drosophila*: use of intron spacers.. Methods.

[40] Jin P, Zarnescu DC, Zhang FP, Pearson CE, Lucchesi JC (2003). RNA-mediated neurodegeneration caused by the fragile X premutation rCGG repeats in *Drosophila*.. Neuron.

[41] Tully T, Preat T, Boynton SC, Delvecchio M (1994). Genetic Dissection of Consolidated Memory in *Drosophila*.. Cell.

[42] Coyle P, Zalewski PD, Philcox JC, Forbes IJ, Ward AD (1994). Measurement of zinc in hepatocytes by using a fluorescent probe, Zinquin: relationship to metallothionein and intracellular zinc.. Biochemical Journal.

