# Supplementary figures and images for: Genetic Inhibition of Solute-Linked Carrier 39 Family Transporter 1 Ameliorates Aβ Pathology in a Drosophila Model of Alzheimer's Disease
Source: PLoS Genet. 2012 Apr 26;8(4):e1002683. doi: 10.1371/journal.pgen.1002683 (PMC3343105; doi:10.1371/journal.pgen.1002683)

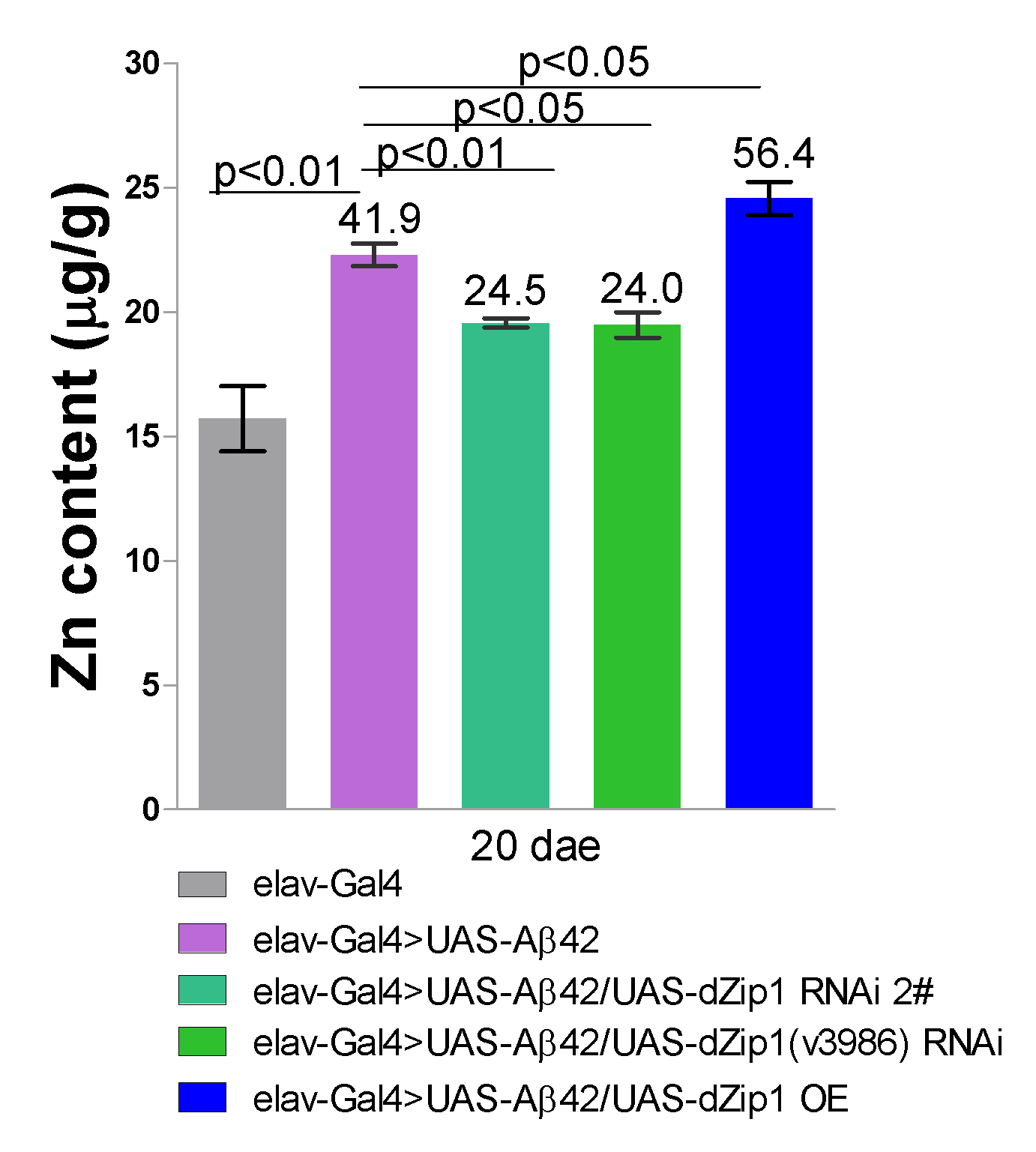

Supplement: Figure S1 — 20-day old Drosophila brain zinc levels are affected by Aβ42 and dZip1 expression. Zn content in brains of 20-day old flies was measured by ICP-OES. dZip1 OE (elav-Gal4>UAS-Aβ42/UAS-dZip1) facilitated Zn accumulation in fly brains compared with control Aβ42 flies (elav-Gal4 vs. elav-Gal4>UAS-Aβ42). dZip1 RNAi slowed down the brain Zn accumulation process (elav-Gal4>UAS-Aβ42/UAS-dZip1-RNAi 2# and elav-Gal4>UAS-Aβ42/UAS-dZip1(v3986) RNAi). Data on top of the bars represent the relative increased percentage of Zn level to control elav-Gal4>+/+ flies. Data are expressed as means ± SEM and analyzed by Student's t-test. n = 3 for each genotype. (TIF) [file pgen.1002683.s001.tif]

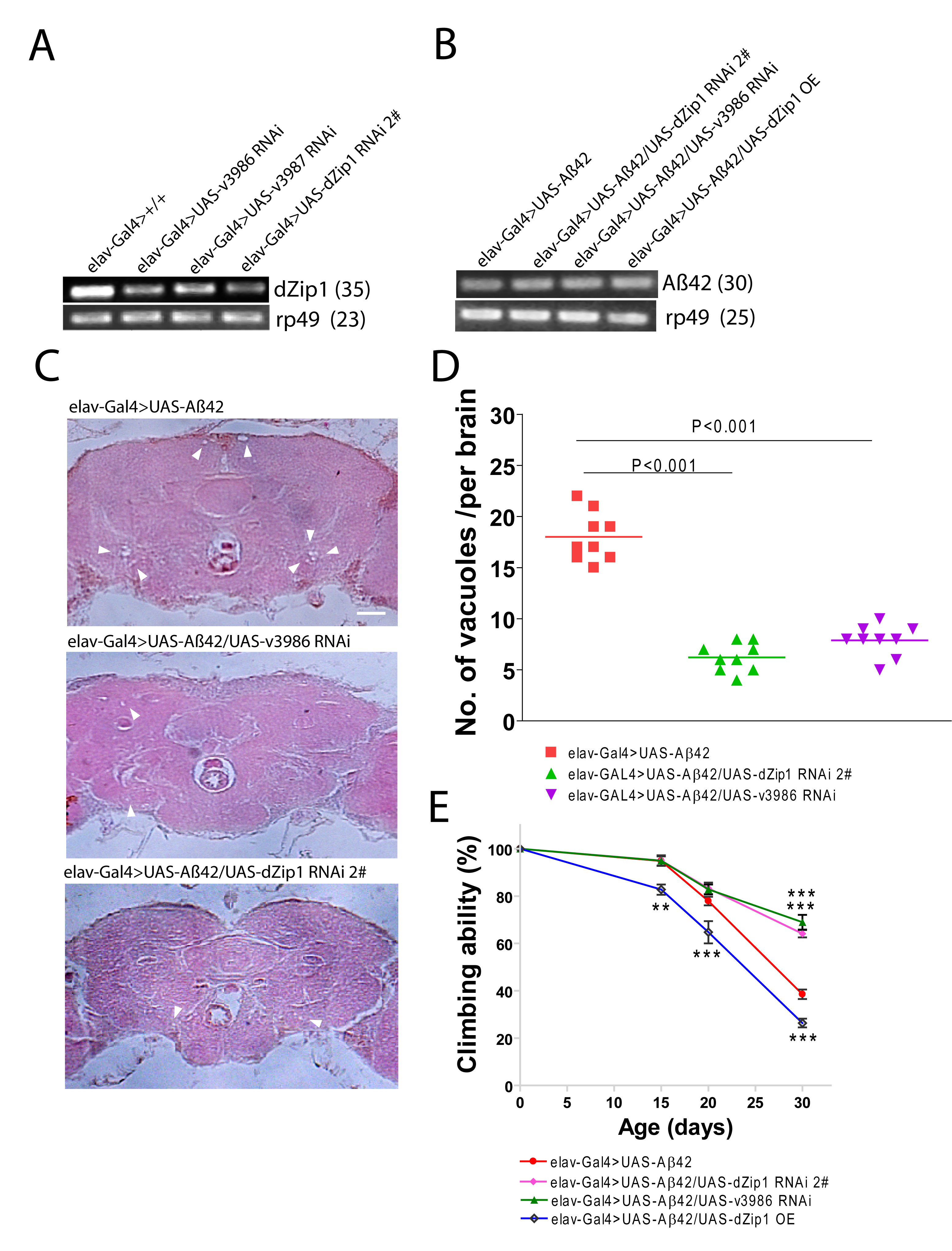

Supplement: Figure S2 — V3986, an independent dZip1 RNAi line, could similarly rescue Aβ-associated brain vacuolization and climbing defect. (A) shows a representive of sqRT-PCR analysis of dZip1 expression in brains of 5-day old flies.V3986 RNAi line shows similar knock down effect with dZip1 RNAi 2#, which is better than v3987 RNAi line. rp49 was used as the loading control. (B) shows a representive of sqRT-PCR analysis of Aβ42 expression in brains of 30-day old flies.V3986 RNAi line shows similar Aβ42 expression level with dZip1 RNAi 2# line and dZip1 OE line, and no reducing Aβ42 expression level was found compared to Aβ42 flies. rp49 was used as the loading control. (C) Paraffin sections of 30-day old fly brains were stained with H&E. V3986 RNAi line produced similar rescuing effect as dZip1 RNAi 2# line on Aβ42 expression induced neurodegeneration (arrowheads indicate the vacuoles). Scale bar, 50 µm. (D) is a statistical analysis of (C) Aβ42-induced neurodegeneration under dZip1 expression modulation. Number of vacuoles (diameter>3 µm) on each section was counted and summarized. V3986 RNAi line produce similar rescuing effect as dZip1 RNAi 2# line, which has significant less vacuole numbers than control elav-Gal4>UAS-Aβ42 flies (p<0.001). Data are expressed as means ± SEM and analyzed by the Student's t-test. n = 9 for each genotype. (E) V3986 RNAi line has similar rescuing effect as dZip1 RNAi 2# line on Aβ42 expression induced climbing defect. t-test, **P<0.01, ***P<0.001 (in comparison with elav-Gal4>UAS-Aβ42 flies). n = 6 independent experiments. (TIF) [file pgen.1002683.s002.tif]

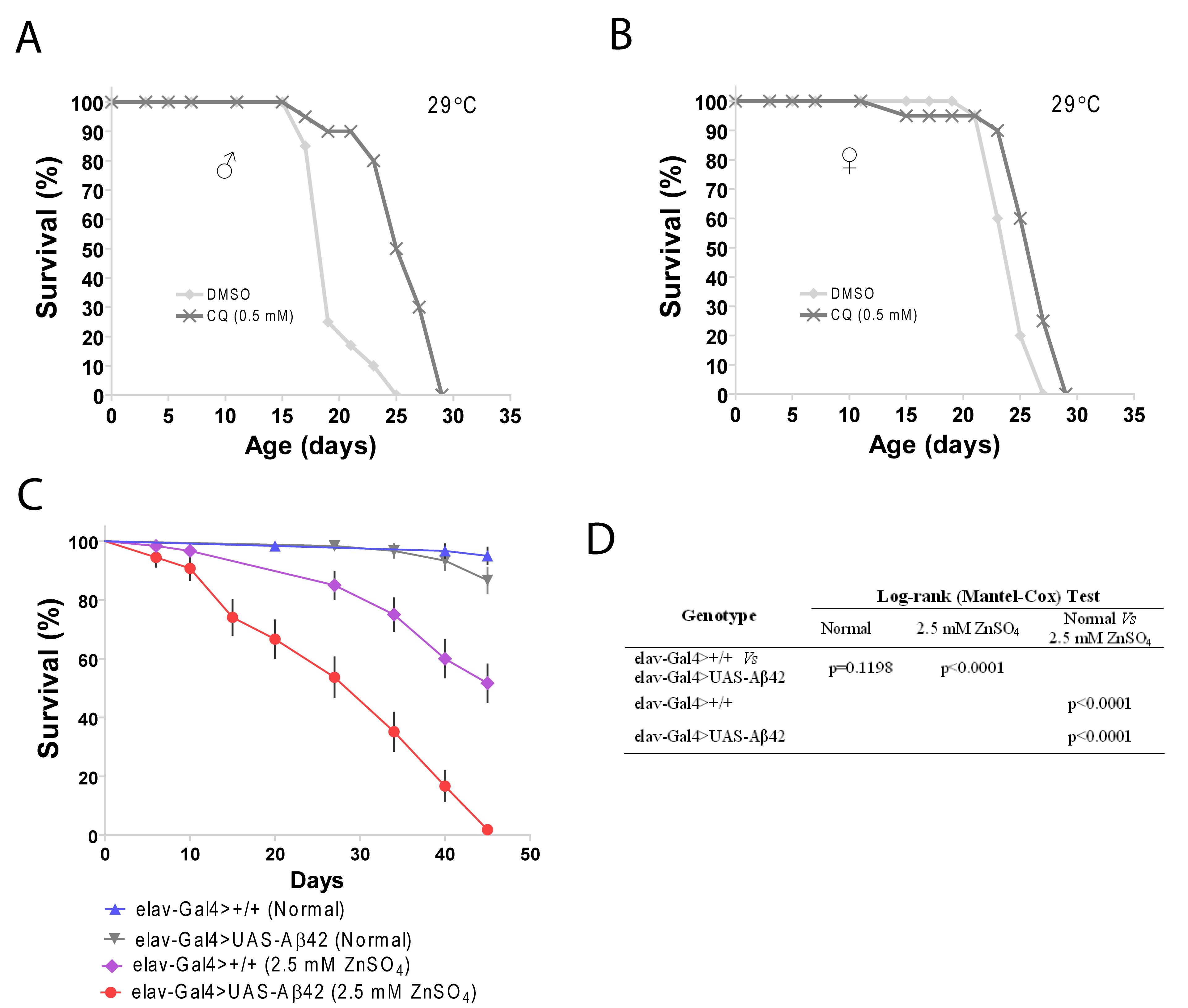

Supplement: Figure S3 — Zinc chelation with clioquinol extends while zinc addition shortens Aβ42 survival. Male (A) and female (B) elav-Gal4>UAS-Aβ42 flies were raised on normal food with DMSO (control) and normal food supplied with 0.5 mM clioquinol (CQ) at 29°C. (C) shows the survival curves of elav-Gal4 and elav-Gal4>UAS-Aβ42 flies raised on normal food or supplied with 2.5 mM ZnSO4, at 25°C. (D) was a Mantel-Cox log-rank statistical analysis of (C) survival curves. The percentage of survivorship was plotted against the age (dae). Foods were changed every 2–3 days, and the survival numbers of flies were counted. At least three biological repeats were used for each genotype. (TIF) [file pgen.1002683.s003.tif]

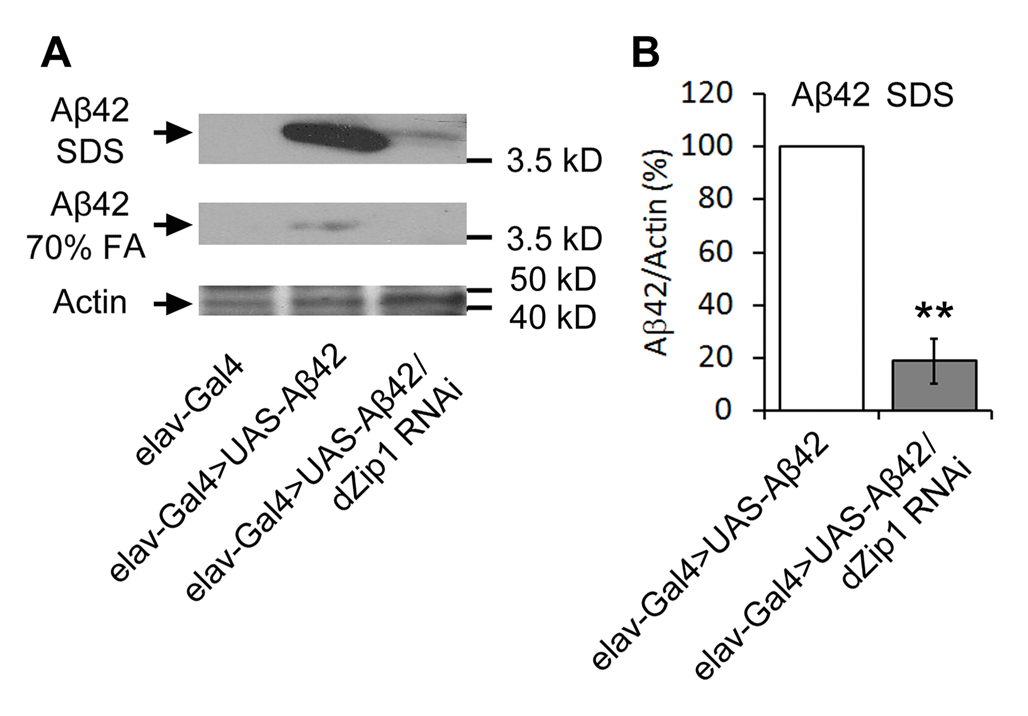

Supplement: Figure S4 — dZip1 expression reduction decreases both the low and high aggregated forms of Aβ42. Protein lysates from equal number of 30-day old fly heads were prepared for western blotting assay. (A) shows a representative of western blot experiments. No Aβ42 was detected in control elav-Gal4 flies. SDS-soluble and SDS-insoluble but formic acid-soluble Aβ42 were detected in elav-Gal4>UAS-Aβ42 flies. Less SDS-soluble Aβ42 and no formic acid-soluble Aβ42 were found when dZip1 expression was inhibited by RNAi. (B) Statistical analysis of SDS soluble Aβ42 bands in (A). t-test, **P<0.01 (in comparison with elav-Gal4>UAS-Aβ42 flies). n = 3 independent experiments. (TIF) [file pgen.1002683.s004.tif]

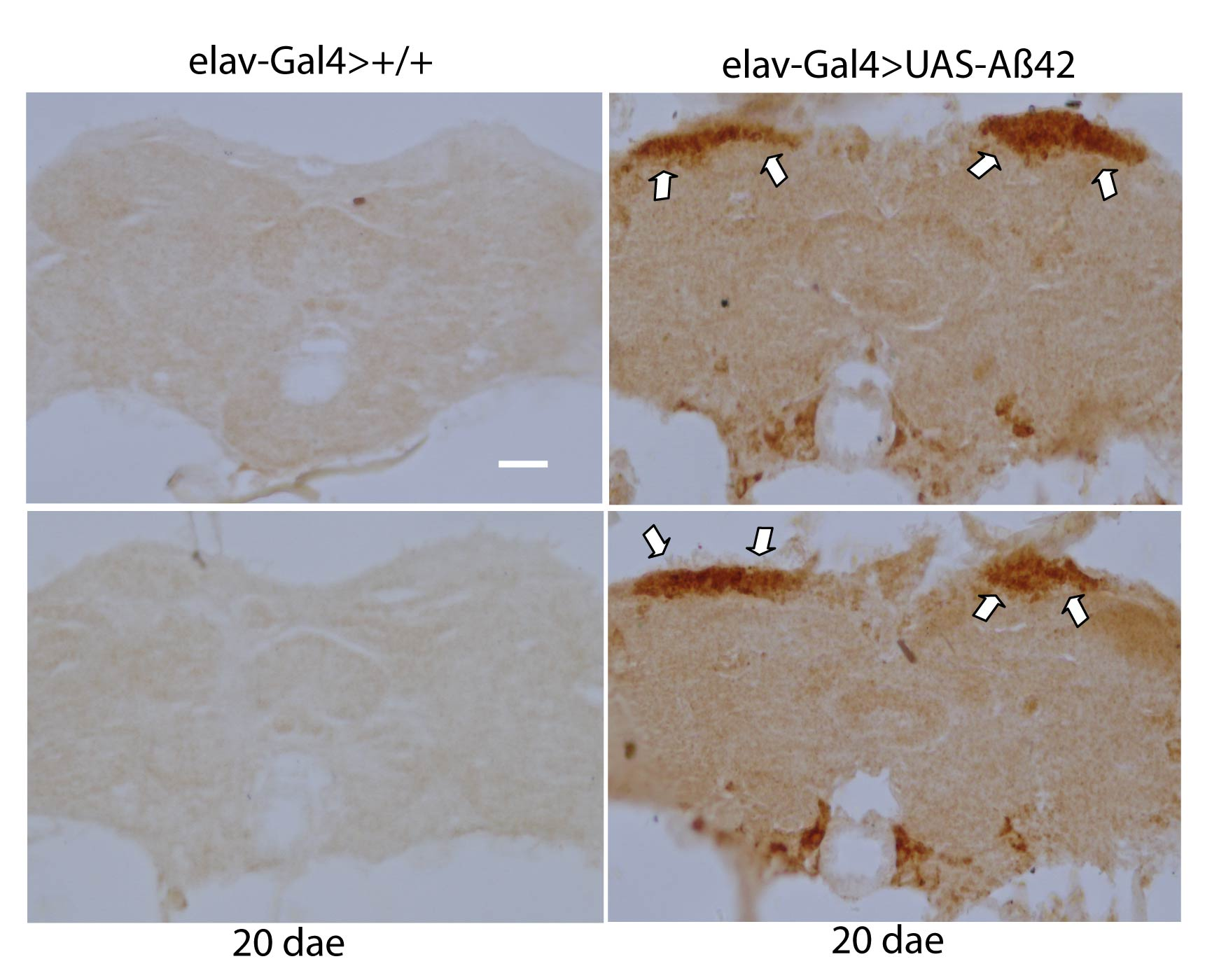

Supplement: Figure S5 — The Aβ42 antibody is specific to Aβ42 in the fly brain. Paraffin sections of 20-day old elav-Gal4 and elav-Gal4>UAS-Aβ42 fly brains were stained with antibody against Aβ42. Pan-neuronal expression of Aβ42 in fly brains induced Aβ42 accumulation which was primarily located in the neuronal somatic region (arrow). No Aβ42 signals were detected in age-matched control brains without Aβ42 expression. Scale bar, 50 µm. (TIF) [file pgen.1002683.s005.tif]
