# Supplementary material for: Genetic Inhibition of Solute-Linked Carrier 39 Family Transporter 1 Ameliorates Aβ Pathology in a Drosophila Model of Alzheimer's Disease
Source: PLoS Genet. 2012 Apr 26;8(4):e1002683. doi: 10.1371/journal.pgen.1002683 (PMC3343105; doi:10.1371/journal.pgen.1002683)
Supplement: Table S1 — Real-time RT–PCR primers used in this study. (DOC) [file pgen.1002683.s006.doc]

Table S1 Real time RT-PCR primers used in this study

| **Gene name** | **Primer sequence (Forward)** | **Primer sequence (Reverse)** |
| --- | --- | --- |
| dZip1 | 5’-GGTGGTGCGGTGCCTACTCTTTT-3’ | 5’-TTCCTTCGCCTGTGGATTGAGG-3 |
| NEP1 | 5’-GATGACGCAGGGCGAGAA-3’ | 5’-TGGGCGTAGTTGAGAAAGAACA-3’ |
| NEP2 | 5’- CCGCAGATGGGCTGAGAA-3’ | 5’-TGCACGCCGGTAGTAATACG -3’ |
| NEP3 | 5’-GTCCAGCCGCACCAAAAA-3’ | 5’-CCATTGATTGCAGGAATATCCA-3’ |
| IDE | 5’-AAAGAGGGACCCAAGAAGTG-3’ | 5’- ATATTTGCATGGACGAGACG-3’ |
| Aβ42 | 5’-CCGACATGACTCAGGATAT-3’ | 5’-TATGACAACACCGCCCAC-3’ |
| rp49 | 5’- TACAGGCCCAAGATCGTGAA-3 | 5’- TCTCCTTGCGCTTCTTGGA-3 |
